# Isolation of fungi using the diffusion chamber device FIND technology

**DOI:** 10.3762/bjoc.15.216

**Published:** 2019-09-19

**Authors:** Benjamin Libor, Henrik Harms, Stefan Kehraus, Ekaterina Egereva, Max Crüsemann, Gabriele M König

**Affiliations:** 1 Institute for Pharmaceutical Biology, University of Bonn, Nussallee 6, 53115 Bonn, Germany

**Keywords:** FIND, fungal one-step isolation device, *Heydenia* cf. *alpina*, natural products, terpenes

## Abstract

Fungi are an important source of bioactive metabolites. The Fungal one-step IsolatioN Device (FIND) technology allows the isolation of rare fungi from terrestrial and marine samples. The FIND comprises a multi-chambered micro agar plate, where initially only one fungal part (e.g., hyphal cell, mycelial fragment or spore) is located in each chamber. After inoculation the device is placed back into the original natural environment of sample collection, to ensure favourable growth conditions. Experiments were carried out with terrestrial soil and marine sediment, as well as sea water samples to validate this method. This yielded axenic cultures of 12 different filamentous fungi, one of them being the marine-adapted fungal strain *Heydenia* cf. *alpina*. The latter produced two new terpenoids, which are the first secondary metabolites from this genus.

## Introduction

Natural products play a prominent role as lead structures in drug discovery [1]. Apart from bacteria, fungi are an impressive group of microorganisms in this respect, due to the high structural diversity of their rich secondary metabolism. Fungi are known as producers of many therapeutically important drugs, such as antibiotics (e.g., cephalosporins), immunosuppressants (e.g., mycophenolic acid) and immunomodulators (e.g., fingolimod), as well as cholesterol lowering agents (statins). Generally, fungal metabolites were shown to have antiviral, cytotoxic, antineoplastic, cardiovascular, anti-inflammatory, immune-stimulating, and anticancer activities [2]. Since the World Health Organization (WHO) declared antibiotic resistance a global threat and urged the search for new antibiotics, the interest in fungal metabolites increased recently.

In order to find new structural types, novel source organisms have to be targeted. It is however, most difficult to isolate unusual, to date not yet researched microbes in axenic form and to cultivate them. Overall it was estimated that only 1% of all microbes present in our environments are cultivated today (“great plate count anomaly”) [3]. Recently the new bacterial species *Eleftheria terrae*, alongside other new microbial species, was isolated from an environmental sample with a device known as iChip. This technology is based on the incubation of a single bacterial cell in its natural environment. After this incubation, strains can be transferred as axenic cultures to the laboratory [4]. *E. terrae* produced a novel antibiotic called teixobactin with no detectable resistance, as it interacts with key functions of cell wall synthesis that do not undergo fast evolutionary changes [5].

Inspired by this approach we developed the Fungal one-step IsolatioN Device (FIND), in order to obtain axenic cultures of rare and less examined filamentous fungi, as several tries before with the iChip were not successful. Apart from terrestrial soil probes we focused on the marine habitat, probing sediment and sea water samples for the presence of fungi. With 150 000 species named and classified, it was suggested that 95% of fungi to date remain unknown [6]. The so-called dark matter fungi (DMF) are a promising source for novel compounds [7–10] and their isolation and culture is one of the keys to find new pharmacological agents.

## Results

### Isolation of axenic fungal strains using FIND

For the isolation of fungal strains from terrestrial and marine environments a device was constructed, i.e., FIND that is similar to the one described by Epstein et al. for “unculturable” bacteria [4]. For the FIND we adjusted the isolation procedure and technical features, e.g., the dimensions of the through holes utilized as growing chambers, to fungi. The FIND protocol now suits fungal physiology and the prevalence of fungi in different habitats.

**Scheme 1:**
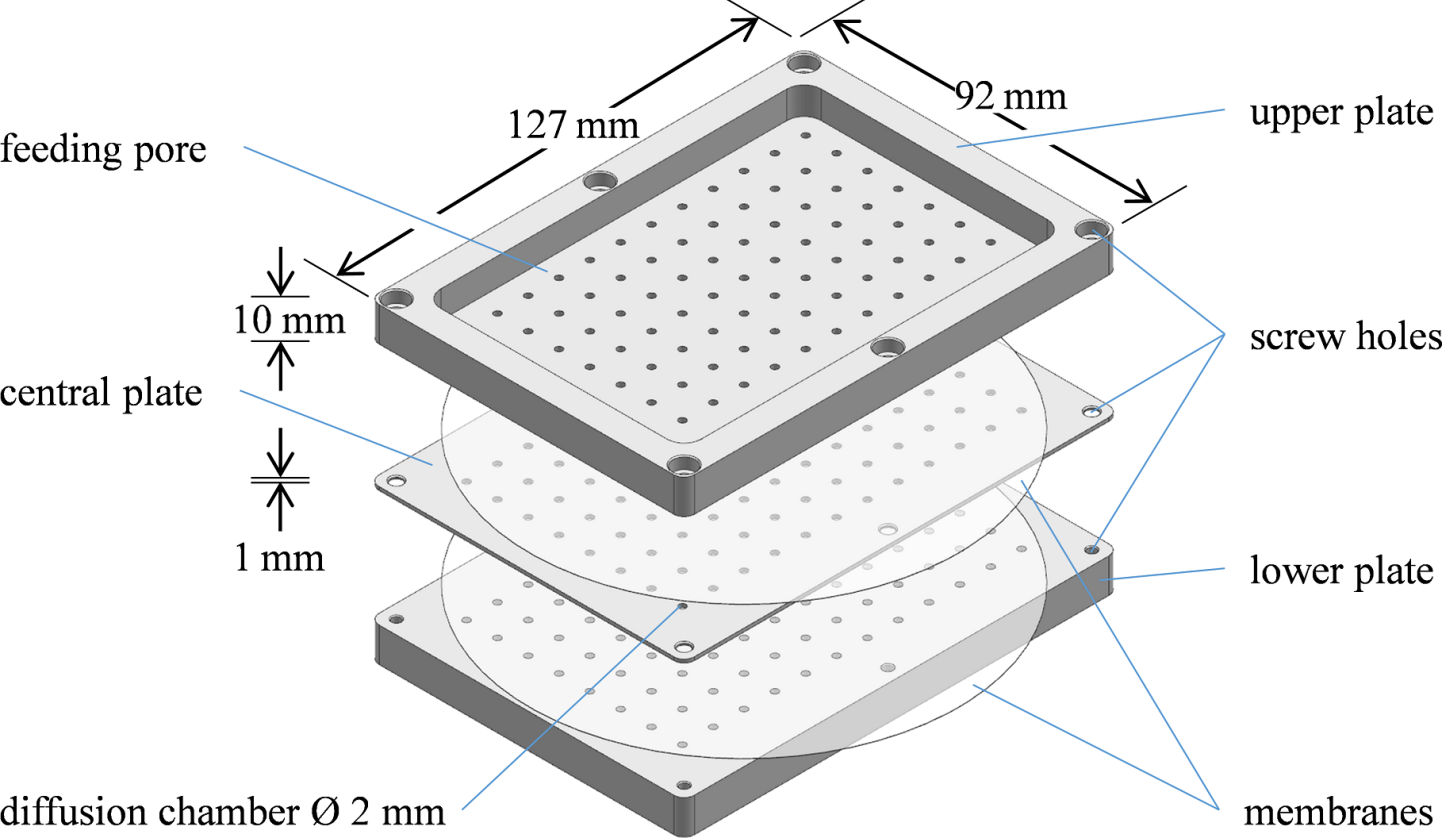
Design and functional parts of the FIND technology.

In general, the FIND technology is a multichambered micro agar plate. After inoculation, the device is placed back into the original natural environment of sample collection, to ensure favourable growth conditions for specific fungi. Inoculation is performed in only one step and thus suitable for high throughput approaches. Scheme 1 illustrates the FIND, consisting of merely three plastic parts crafted from polyoxymethylene, which are solid and durable enough for use in most natural environments. The central plate is a thin plastic board with 96 through holes that function as compartments, capable of holding inoculated agar plugs. Semipermeable membranes are attached to both sides of this plate, this way covering the through holes and preventing contamination by other microbes. These membranes, at the same time, permit diffusion of nutrients and growth factors from the environment through the feeding pores of the upper and lower plates. This way slow growing fungi or to date uncultured fungi can be isolated.

To test the FIND technology, we used samples from terrestrial and marine environments, i.e., soil from Wuppertal, Germany (experiment 1), marine sediments from the Aegean Sea, Greece (experiment 2), brackish soil samples from the Schlei region around Kappeln, Germany (experiment 3), and sea water samples from Katwijk aan Zee, Netherlands (experiment 4).

**Scheme 2:**
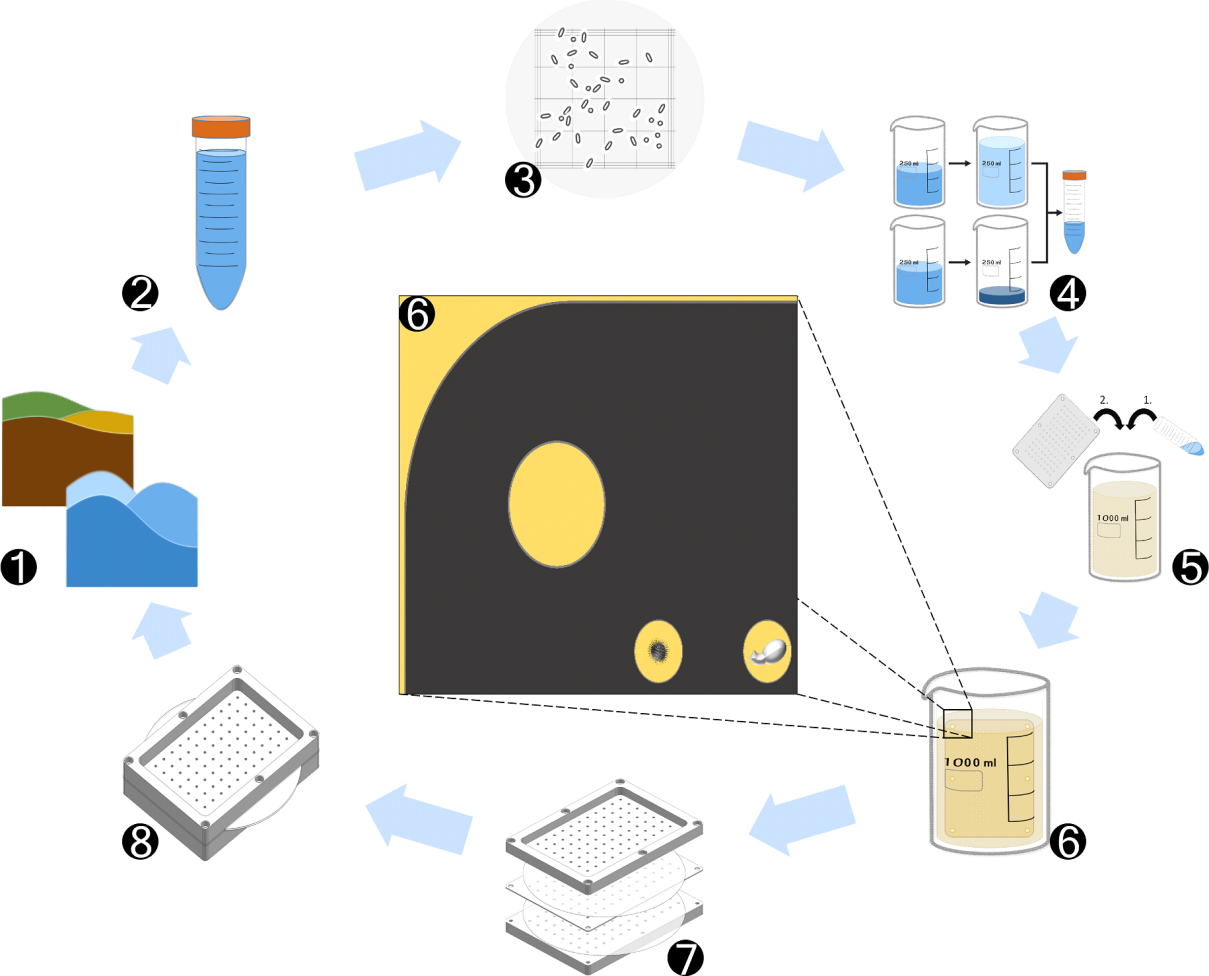
Isolation of fungal strains with the FIND technology. 1. Collection of terrestrial or marine sample. 2. Sample preparations. 3. Determination of the number of microbial cells. 4/5. Dilution or concentration of sample and merging with molten agar. 6. Adjustment of the concentration of fungal parts in such a way that in average one fungal part is finally placed in one growing chamber. 7. Attachment of semipermeable membranes to the central plate and sealing with upper and lower plates. 8. Placement of the loaded and assembled device into the original environment for incubation.

Adjustment of the sample concentration (i.e., number of fungal parts per volume), which is crucial for the success of a FIND experiment, has to take the abundance of fungal parts, i.e., hyphae, mycelial fragments or spores in the collected environmental samples into account. Ideally, for incubation one fungal part per through hole should be present. Thus, for the here presented FIND with 96 through holes each with a volume of 3 × 10^−3^ mL, a concentration of 320 000 fungal parts/L must be reached in the agar solution, with which the central plate is loaded. The number of fungi in a given volume of a sample is experimentally hard to determine. We thus counted bacterial cells, and applied the ratio of bacteria-to-fungi, which is described in the literature for most environments, to estimate suitable dilution or concentration steps in our experiments. Scheme 2 illustrates each individual step of a FIND experiment.

For experiment 1 (terrestrial soil sample) a small volume of soil was treated with a sterile-filtered isotonic NaCl solution [11–13]. After centrifugation cell counting was performed on the supernatant using a Neubauer Improved or Thoma chamber [14] to determine the abundance of bacterial cells. For our soil sample 2 ± 0.25 × 10^13^ bacteria/L were determined. For experiment 2 (marine sediment) and 3 (brackish water sediment), samples were treated alike, but instead of a NaCl solution, sea water from the respective sampling site was used. For experiment 4 (sea water), sea water was analysed directly. The extant abundances of bacterial cells/L in experiments 2, 3 and 4 were found to be 7 ± 0.7 × 10^9^, 13 ± 5 × 10^9^ and 8 ± 0.8 × 10^9^ bacterial cells/L, respectively.

The samples were then diluted, concentrated or used undiluted according to the bacteria-to-fungi ratio, in order to ultimately reach an approximate target concentration of 320 000 fungal parts/L. Literature documented a bacteria-to-fungi ratio as 10³ to 1 for terrestrial soil [15]. Thus, the sample from Wuppertal (experiment 1) was diluted sequentially to a concentration of 2 × 10^10^ bacterial cells/L, which should be equivalent to 2 × 10^7^ fungal parts/L, and 16 mL of this solution were necessary to be added to one litre of agar solution to obtain the required target concentration.

For marine sediment samples (experiments 2 and 3), in general, the bacteria-to-fungi ratio was hardly known. Thus, we applied the ratio described for terrestrial soil samples, i.e., 10³ to 1. With abundances of 7 × 10^9^ and 13 × 10^9^ bacterial cells/L concentrations of 7 × 10^6^ and 13 × 10^6^ fungal parts/L, respectively, were assumed. The samples were thus used undiluted and 42 and 24 mL, respectively, were added to one litre of agar solution to obtain the required target concentration. Using this ratio, in our experiments, however, we had hardly any success (apart from one fungal isolate, i.e., *Cladosporium allicinum* and *Heydenia* cf. *alpina* from samples 2 and 3, respectively). Obviously in these experiments the concentration of fungal parts was too low, and thus we addressed this question in experiment 4 more carefully.

Abundances of around 1000 fungi/L sea water were documented for samples from the North Sea [16–17]. Considering this information, we performed experiment 4 on the assumption of an abundance of 1000 fungi/L, which required fungal cells of 8 L of sea water in a volume of 25 mL agar solution in step 5 of the FIND procedure. Since our bacterial count on this unprocessed sea water sample had revealed an abundance of 8 × 10^9^ cells per L, we ourselves calculated a bacteria-to-fungi ratio of around 10^7^ to 1 for subsurface samples from littoral areas of the North Sea. This is in accordance to literature reports for surface slick and subsurface samples from littoral areas of Florida, where ratios of 10^5^ to 1 and higher were determined [18].

Vacuum filtration of the marine water sample (10 L) from Katwijk aan Zee/North Sea through paper disks with a pore size of approx. 2 µm, i.e., wide enough to let pass bacteria, but small enough to concentrate fungal spores and mycelia in the filter cake, was performed. Around 5 mL of filter cake was resuspended in another 5 mL of sterile sea water and mixed with 15 mL agar solution.

In summary, the diluted sample from experiment 1, the undiluted solutions from experiments 2 and 3, and the concentrated, resuspended sea water solution from experiment 4 were each mixed with a warm agar preparation, containing a minimal nutrient medium and antibiotics (experiment 1: amino acids/peptone, antibiotics; experiments 2–4: ditto, but in sea water) to yield the desired concentration of roughly 320 000 fungal parts/L. Then, the central plate of FIND (Scheme 1) was lowered into these preparations to fill each chamber with an agar solution, containing in average one fungal part. As the agar solidified, the fungal spore or mycelium was trapped in the chamber and separated from other ones, e.g., competing organisms. Semipermeable membranes were attached to both sides of the central plate and the device was sealed with the upper and lower parts and fixed with screws. To guarantee that the device was sealed properly, commercial aquarium safe silicone without antifungal additive was applied to the edges. All materials and tools were sterilized by autoclaving or surface disinfection and the procedure was performed under the laminar airflow cabinet.

The sample-loaded and sealed device was then inserted into an appropriate container with 40 L of the original soil for experiment 1, sea water and sediment from the collection sites for experiments 2 and 3, whereas for experiment 4 (sea water) 20 L of sea water from the sample site were used to mimic environmental conditions of the corresponding sample sites.

At this stage the trapped organisms are separated from each other, but connected to their original environment via semipermeable membranes. This way the fungal seeds are provided with growth factors exclusively found in their natural habitat. The device was incubated for four weeks. Deionized water was applied to the soil of experiment 1 every 2 days. An aquarium water pump provided water current simulation and evaporated tank water was replaced by autoclaved deionized water in experiments 2–4.

After incubation the device was removed from the tanks, rinsed with autoclaved deionized water to remove sediment and soil and opened under a laminar air flow cabinet. Each agar plug was transferred to a corresponding agar plate for cultivation. The agar plates contained the same minimal nutrient medium as the agar solution into which the central plate was dipped earlier. Alternatively, a stamp can be used to punch all agar plugs simultaneously into the wells of a 96 well plate. Fermentation on agar plates was performed in an incubator at 25 °C for another four weeks. Morphology of the axenic colonies was then analysed microscopically, and their taxonomy was additionally identified by DNA sequencing.

In total, experiments 1–4 led to 76 fungal isolates (41 fungi in experiment 1, 1 fungus in experiment 2, 1 fungus in experiment 3 and 33 fungi in experiment 4). All fungal cultures were obtained in single isolation steps directly via the FIND procedure and found to be axenic. From experiment 1, 39 isolates were microscopically identified as belonging to genera common in soil, e.g., *Fusarium*, *Penicillium* or *Trichoderma* and thus were not further processed. The addition of antibiotics prevented bacterial contamination in all marine samples. However, a few antibiotic resistant strains were isolated from soil but also not further processed. One isolate from experiment 4 was found to belong to the genus *Cladosporium*, and therefore was also not considered further. The remaining 36 isolates were identified at least to the genus level (Table 1). The pooled results from comparison (BLAST) of DNA sequences (ITS1, ITS2, 18S, 5.8S, 28S, RPBII, tef1α and β-tubulin) with reference sequences deposited in public databases (GenBank) and microscopical analysis of morphological characteristics, identified 12 ascomycetes from 12 different genera. Except for *Scopulariopsis brevicaulis* (25 isolates), all strains were encountered only once, due to the ideally adjusted microbial concentration and homogenisation of the sample material. Nine of those 12 different isolates could be identified to species level, whereas for the remaining three (*Cadophora* sp., *Chrysosporium* sp., *Leucothecium* sp.) no species could be assigned. In the case of fungi of the genus *Cadophora*, genetic information for all 25 hitherto described species (MycoBank) are deposited in GenBank. As the alignments of our sequences with the best matching ones of a cultured species, e.g., *C. orchidicola* reached only 97% identity in BLAST, it can be assumed that our isolate is an undescribed species, as it shows 99% identity with sequences of an undescribed species (Table 1). For the 116 legitimate species of *Chrysosporium*, BLAST of our sequences revealed 99% identity with reference sequences of *Ch. submersum*. Unfortunately, sequences for only 33 *Chrysosporium* species are available as reference sequences, and thus no identification on the species level could be achieved. Interestingly, the sequence of our isolated *Leucothecium* species is 100% identical to the reference sequence of an uncultured fungus isolated from cotton in 2014 and seems to be closely related to the type species *L. emdenii,* which is one of only two known species among *Leucothecium*. Most of the isolates obtained in our experiments belong to the Sordariomycetes, the by far biggest class of ascomycetes, and to the Dothideomycetes. Some of our cultures, however, are from less examined classes, e.g., Eurotiomycetes and Leotiomycetes. Only *Chaetomium globosum* aggr. comes into consideration as a common air mould.

**Table 1:** Fungal taxa obtained by four FIND experiments and identified by sequence comparison with the best BLASTn match within the NCBI GenBank database.

Species/genus [GenBank accession number]	ID%	Number of bp analysed	Taxonomic class

*Clonostachys rosea* (Link:Fries) Schroers et al.^1^ [KT323182]	99^a^	506	Sordariomycetes
*Ilyonectria europaea* A. Cabral, Rego & Crous^1^ [JF735294]	100^b^	531	Sordariomycetes
*Cladosporium allicinum* (Fries) Bensch, U. Braun & Crous^2^ [MH118272; MH567104]	100^c^; 100^d^	511; 241	Dothideomycetes
*Heydenia* cf. *alpina^3^* [KF574887]	100^a^	549	incertae sedis
*Alternaria armoraciae* E.G. Simmons & G.F. Hill^4^ [KC584638]	100^d^	236	Dothideomycetes
*Auxarthron* cfr. *umbrinum* (Boudin) G.F. Orr & Plunkett^4^ [MH857026]	99^b^	509	Eurotiomycetes
*Cadophora* sp.^4^ [FJ820724; KF636777]	99^b^; 99^e^	552; 999	Leotiomycetes
*Chaetomium globosum* aggr.^4^ [MH644079; MG890100]	100^f^; 100^g^	380; 585	Sordariomycetes
*Chrysosporium* sp.^4^ [MF939600]	99^h^	538	Eurotiomycetes
*Leucothecium* sp.^4^ [LT608439]	100^i^	200	Sordariomycetes
*Metarhizium carneum* (Duché & R. Heim) Kepler, S.A. Rehner & Humber^4^ [MH864783]	100^b^	624	Sordariomycetes
*Scopulariopsis brevicaulis* Bainier^4^ [LM652465]	100^b^	609	Sordariomycetes

^1^Eskesberg, Wuppertal (DE), soil sample, ^2^Aegean Sea (GR), marine sediment, ^3^Kappeln (DE), brackish water sediment, ^4^Katwijk aan Zee (NL), sea water ^a^18S rRNA gene, ITS1, 5.8S rRNA gene, ITS2, ^b^18S rRNA gene, ITS1, 5.8S rRNA gene, ITS2, 28S rRNA gene, ^c^ITS1, 5.8S rRNA gene, ITS2, ^d^tef1α, ^e^28S rRNA, ^f^β-tubulin, ^g^RPBII, ^h^ITS1, 5.8S rRNA gene, ITS2 and 28S rRNA gene, ^i^ITS2

In a next series of experiments, we set out to shed light on the halotolerance of our isolates from experiments 2 and 3 by testing their salt tolerance or dependency. Thus, we analysed the growth behaviour of *Cladosporium allicinum* and *Heydenia* cf. *alpina* on agar plates containing growth medium of increasing salinity from 0–35‰ (sea water has a salinity of approx. 35‰). As outcome the diameter of radial growth was measured after 14 days. All experiments were performed in triplicate on 130 mm agar plates containing bio malt agar medium. For the isolate from the Mediterranean Sea (experiment 2), i.e., *C. allicinum*, our results showed optimal growth conditions at 35‰ salinity, i.e., a diameter of 107 ± 2 mm was reached. Only poor growth of 54 ± 1 to 89 ± 1 mm was observed at levels of salinity of 14‰ and below. For the isolate from brackish water (experiment 3), i.e., *Heydenia* cf. *alpina* levels of salinity above 7‰ were necessary to achieve an adequate growth of 124 ± 1 to 130 ± 0 mm. Below 7‰ salinity the radial growth rates of latter cultures dropped dramatically to only 67 ± 1 mm in diameter. In both cases growth rates were nearly halved when the growth medium was lacking appropriate concentrations of sea salts. Our results show that these fungi require high salt concentrations for optimal growth, and this suggests an adaption of the respective strains to marine conditions (see Supporting Information File 1, Tables S1 and S2 for full experimental data).

With the FIND technology we managed to isolate rare fungi. To evaluate the potential for the biosynthesis of bioactive secondary metabolites we performed screenings on antimicrobial activities against Gram-negative and Gram-positive bacteria, an ascomycete, a basidiomycete and a zygomycete, i.e., *Escherichia coli*, *Bacillus megaterium, Eurotium rubrum*, *Microbotryum violaceum* and *Mycotypha microspora*. Ethyl acetate extracts of *C. rosea*, *I. europaea* grown on BM medium and of the other 10 isolates grown on BMS medium were tested in disk diffusion assays using 50 µg of extract per disk. None of the extracts was active against the Gram-negative bacterium *E. coli.* Extracts of *A. armoraciae, Auxarthron umbrinum, C. globosum, Chrysosporium* sp. 831*, C. allicinum, C. rosea, H.* cf. *alpina* and *I. europaea* inhibited the growth of *B. megaterium*, i.e., inhibition zones of 1, 2, 1.5, 1, 4, 5, 5 and 3 mm were reached, respectively (streptomycin, equally concentrated, was used as positive control with an inhibition zone of 10 mm). Additionally, the extract of *I. europaea* inhibited the growth of *M. violaceum* with an inhibition zone of 3 mm in diameter (miconazole, half concentrated, was used as positive control with an inhibition zone of 10 mm; see Supporting Information File 1, Table S3 for full experimental data).

### Detailed chemical investigation of *H*. cf. *alpina* including structure elucidation of the new metabolites **1** and **2**

On the basis of its preferred growth on saline media, the *H.* cf. *alpina* (strain No. 824), isolated during this study, was selected for further chemical investigations. To the best of our knowledge this is the first chemical investigation of a member of the *Heydenia* genus.

From the solid, sea-salt containing cultures of *H.* cf. *alpina* the ethyl acetate soluble organic compounds were analysed. A first fractionation was achieved by vacuum liquid chromatography (VLC) on reversed-phase material yielding 4 fractions. ^1^H NMR analysis of these indicated the presence of chemically diverse secondary metabolites in the major VLC fraction 3. Detailed HRESIMS investigation was thus performed with VLC fraction 3, which showed prominent *m/z* values for metabolites with molecular weights of 248 and 250 Da.

**Figure 1:**
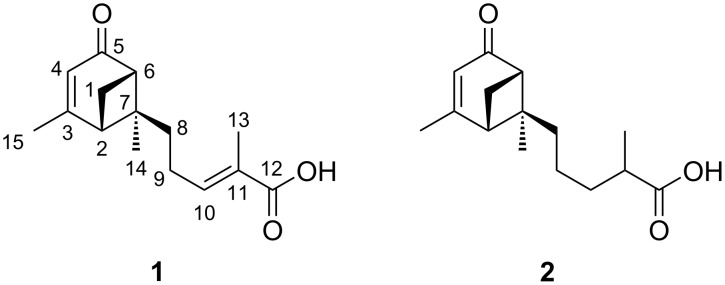
Secondary metabolites isolated from *H.* cf. *alpina*.

Subsequent repeated fractionation of VLC fraction 3 by RP-HPLC resulted in the isolation of two, structurally closely related, new secondary metabolites, **1** and **2** (Figure 1).

Compound **1** was obtained as a yellow oil with a molecular formula of C_15_H_20_O_3_, implying 6 degrees of unsaturation (DOU), as deduced by HRESIMS (*m/z* 249.1481 [M + H]^+^; calcd. for C_15_H_21_O_3_, 249.1485). The IR spectrum displayed an absorption band at 1670 cm^−1^ indicating the presence of an α,β-unsaturated carbonyl moiety [19].

The ^1^H NMR spectrum (Table S4, Supporting Information File 1) exhibited characteristic singlets for three methyl groups, as well as two olefinic protons, together with the MS data pointing towards a terpenoid like structure. The ^13^C NMR and DEPT-135 spectra of **1** (Table S4, Supporting Information File 1) displayed 15 carbon signals for three methyl, three methylene, two sp^3^ methine and two sp^3^ quaternary carbons. Four characteristic shifts in the ^13^C NMR spectrum at δ_C_ 174.1 (C-3), 122.1 (C-4), 143.0 (C-10) and 129.5 (C-11) evidenced two carbon–carbon double bonds. Together with a carbonyl carbon at δ_C_ 206.6 and a carboxylic carbon at δ_C_ 177.8, the two remaining DOU were accounted to a bicyclic ring system.

**Figure 2:**
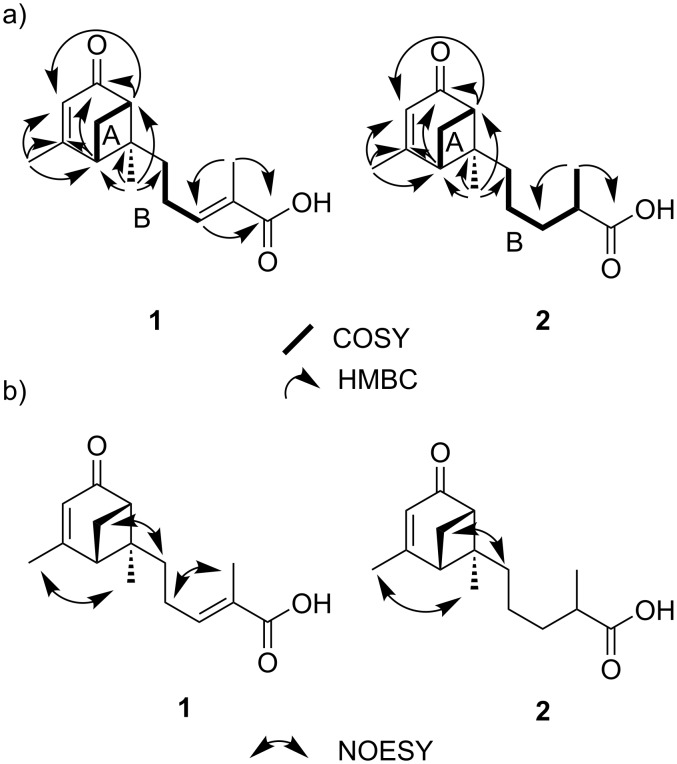
a) Significant ^1^H,^1^H-COSY and ^1^H,^13^C-HMBC correlations for compounds **1** and **2**. b) Key NOESY correlations for compounds **1** and **2**.

After assigning the proton resonances to the ^13^C resonances of directly attached carbon atoms with an HSQC experiment, analysis of the ^1^H,^1^H-COSY data led to the identification of two major ^1^H–^1^H spin systems, one corresponding to the H-2–H-1_b_–H-6 moiety (spin system A) and the second to the H_2_-8–H_3_-13 subunit (spin system B; Figure 2).

Spin system B was extended by a carboxylic group as indicated by ^1^H,^13^C-HMBC correlations from both H-10 (δ_H_ 6.86) and H_3_-13 (δ_H_ 1.89) to carbon C-12 (δ_C_ 177.8), and identified the partial structure 2-methylpentenoic acid. HMBC correlations between the resonances of H-6 (δ_H_ 2.73) to both C-4 (δ_C_ 122.1) and C-5 (δ_C_ 206.6), between H_3_-15 (δ_H_ 2.10) and C-2 (δ_C_ 49.8), C-3 (δ_C_ 174.1) and C-4 (δ_C_ 122.1), and between H_2_-1 (δ_H_ 2.12; 2.93) and both C-3 (δ_C_ 174.1) and C-5 (δ_C_ 206.6) placed the methyl group CH_3_-15 at carbon C-3 (δ_C_ 174.1), and proved the positions of the carbonyl group C-5 (δ_C_ 206.6) and the double bond ∆^3,4^ in the molecule, as shown in Figure 2. The second partial structure was thus a cyclohexenone ring. The proton resonances of H-2 (δ_H_ 2.65) and H-6 (δ_H_ 2.73) appear as a characteristic triplet of doublets caused by a vicinal coupling with proton H-1_b_ (δ_H_ 2.93; *J* = 6.0 Hz), an unusual large “W” coupling to each other (*J* = 6.0 Hz) as well as an allylic coupling to H-4 (δ_H_ 5.78; *J* = 1.3 Hz). These couplings can only be explained with the rigid structure of **1** supporting the presence of a bicyclo[3.1.1] ring system resembling that of verbenone [20].

HMBC correlations of H_3_-14 (δ_H_ 1.07) to C-2 (δ_C_ 49.8), C-6 (δ_C_ 57.2), C-7 (δ_C_ 58.6) and C-8 (δ_C_ 38.2) confirmed the presence of a verbenone moiety and connected the methylpentenoic acid moiety to the quaternary carbon C-7, completing the planar structure of **1**. Compound **1** is thus a bergamotene type sesquiterpene, containing a bicyclo[3.1.1] ring system as well as a 2-methylpent-2-enoic acid moiety, accounting for all ring double bond equivalents (Figure 2).

The relative configuration of **1** at C-2, C-6 and C-7 was established by a NOESY spectrum. NOESY correlations between H-1_b_ (δ_H_ 2.93) and H_2_-8 (δ_H_ 2.07) indicated that these protons were on the same side of the molecule and together with the NOESY correlation between H_3_-14 and H_3_-15 the relative configurations of C-2, C-6 and C-7 could thus be delineated as 2S*, 6S* and 7S*, respectively.

The absolute configurations of C-2, C-6 and C-7 were determined by comparison of our experimental with simulated circular dichroism (CD) spectra. The latter ones were performed by Wu et al. using (2*S*,6*S*)-verbenone and (2*R*,6*R*)-verbenone and can be applied here, because of the assumed minor effect of the side chain on the CD spectrum [21]. Experimental data for **1** showed a negative Cotton effect in the range of 320–350 nm (321 nm), which suggested according to the calculated CD spectrum of Wu et al. (321 nm) that the absolute configurations of C-2, C-6 and C-7 in **1** are 2*S*, 6*S*, and 7*S*, respectively (see Supporting Information File 1, Figure S2 for the CD spectrum of **1**).

The configuration of the double bond ∆^10,11^ could be determined as *E* by NOESY correlations between proton signals H_2_-9 (δ_H_ 2.32) and H_3_-13 (δ_H_ 1.89). This was confirmed by the strong shielding in the ^13^C NMR spectrum of carbon C-13 typically occurring in double bonds with *E* configuration [22]. Therefore, the structure was established as (*E*)-2-methyl-5-((1*S*,5*S*,6*S*)-2,6-dimethyl-4-oxobicyclo[3.1.1]hept-2-en-6-yl)pent-2-enoic acid, for which the trivial name heydenoic acid A is suggested. Notable is the occurrence of peaks containing *m/z* values attributable to isomers of the isolated metabolite.

Compound **2** has a molecular formula of C_15_H_22_O_3_, (HRESIMS *m/z*: 251.1641 [M + H]^+^; calcd. for C_15_H_23_O_3_, 251.1642) implying only 5 DOU as compared to **1**.

^1^H and ^13^C NMR data (Table S5, Supporting Information File 1) of **2** were similar to those of **1**, except for the chemical shifts of CH_2_-9 (δ_H_ 1.37; δ_C_ 23.4), CH_2_-10 (δ_H_ 1.49, 1.73; δ_C_ 35.5), and CH_3_-13 (δ_H_ 1.21; δ_C_ 17.8). Additional proton signals for H-11 and H_2_-10 (δ_H_ 2.50; H-11, δ_H_ 1.49 and δ_H_ 1.73; H_2_-10) proved that **2** is a derivative of **1**, in which ∆^10,11^ is saturated. COSY and HMBC data of **2** fully supported this assumption. NOESY correlation between H_b_-1 (δ_H_ 2.89) and H_2_-8 (δ_H_ 1.94) indicated the relative configurations of C-2, C-6 and C-7 to be the same as in **1**. The CD spectrum of **2** showed also a negative Cotton effect as **1**, indicating the absolute configurations of C-2, C-6 and C-7 to be all *S* (see Supporting Information File 1, Figure S3 for the CD spectrum of **2**). The configuration at C-11 could not be determined. Thus, the structure was established as 2-methyl-5-((1*S*,5*S*,6*S*)-2,6-dimethyl-4-oxobicyclo[3.1.1]hept-2-en-6-yl)pentanoic acid, named as heydenoic acid B.

Compounds **1** and **2** were also tested in antimicrobial assays and showed no effect.

## Discussion

Our experiments demonstrated the potential of the FIND technology for the isolation of fungi from various habitats including terrestrial, littoral and marine areas. We developed an isolation method that is straight forward to use, rapidly performed and provides interesting isolates in a single experiment. In contrast to other isolation methods, e.g., direct plating or dilution plating, where the former can be experimentally difficult and the latter needs several days for colony counts, the FIND procedure is less labour intensive and the count can be performed in a few minutes. A strain of *A*. *umbrinum* was isolated by Alvi et al. using dilution plating on a soil sample [23]. The sample was therefore diluted and spread over the plates and incubated for colony growth. To obtain plates for colony picking several dilutions have to be prepared and inoculated plates have to be incubated only to use a fraction of them for further processing. More challenging is the contamination by fast growing fungi that additionally suppress growth of other species on the same plate. The advantage of FIND lies in the adjustment of the correct dilution before incubation on the one hand and the spatial separation which allows slow growing fungi to form axenic colonies in a single step on the other hand. In times of 3D printers, the device may also be constructed in such a manner. In conventional methods we often experience a mix of species in one colony, which is hard or impossible to separate. In FIND, however, all collected colonies were found to be axenic and ready to be cultivated for further purposes like taxonomic identification or natural product isolation. Isolation methods like baiting may lead to bait-specific fungi only. A strain of *A*. *umbrinum* was isolated by Deshmukh et al. using a hair baiting technique. The majority of those isolates showed keratinolytic activity which proves the bias towards bait-specific fungi [24]. In FIND, fungi regardless of nutritional preferences can be obtained, as they are cultured within the respective environment. In this respect, the most striking feature of the FIND technology is the possibility to reconnect the isolates with their environment, in order to exchange nutrients and growth factors and therefore to ensure species-specific parameters.

An important feature to know before inoculating the FIND is the concentration of fungal parts in the collected environmental sample. Our experiments and literature data showed that the abundances of fungi or fungal parts, i.e., hyphal cells, mycelial fragments or spores, in terrestrial soil samples (2 × 10^9^ fungal parts/L) is huge compared to those in marine sediments (5.000–25.000 fungal parts/L). Only extremely small numbers of fungal parts can be found in sea water (0.3–1000 fungal parts/L) [15–17,25]. These findings together with the high bacteria-to-fungi ratios described in the literature [18] explain the low recovery of fungal strains of experiments 2 and 3. To improve the outcome of experiments performed on sea sediment samples we propose using tenfold of the reported sediment and corresponding sea water in step 2 of the FIND procedure. Additionally, it is crucial to apply the herein described concentration protocol (step 4) and to perform the experiment in a volume as small as possible, i.e., 25 mL to achieve the target concentration (step 5).

Using FIND we isolated and identified 12 ascomycetes belonging to 4 different taxonomic classes (Table 1) from 3 different habitats. For half of these species, e.g., *A. armoraciae, C. allicinum, H.* cf. *alpina, I. europaea* and *M. carneum* to date no secondary metabolites are described in the literature. *A. armoraciae and C. allicinum* are special within their genus, as both belong to chemically underexamined clades, e.g., *Chalastospora* clade and *Cladosporium herbarum* complex, respectively [26–27]. For *C. allicinum* our experiments point towards a salt dependency of growth, i.e., an adaption to the marine environment. Halotolerance was to date exclusively reported for members of the *Cladosporium sphaerospermum* complex [28].

Analysis of genes involved in secondary metabolite production suggested *Cadophora malorum* as a producer of bioactive compounds [29], as later confirmed by the results of Almeida et al. [30], which highlights FINDs potential in the isolation of bioactive compound producing strains. Using FIND enabled the isolation of rare species of the genus *Chrysosporium*, which are scarcely found in soil [31] and of which the world register of marine species only lists four entries, e.g., *C. corda*, *C. pannorum*, *C. magnasporum* and *C. oceanitesii* [32]. With *Leucothecium* sp. a rare as well as fastidious fungus was isolated in this study, where cultivation was reported to be difficult for at least one of the two species belonging to this genus. *L. coprophilum* showed poor growth when cultured under laboratory conditions as it was lacking interactions to certain bacteria coexisting in its natural habitat [33]. In contrast to this, there were no difficulties regarding isolation or cultivation using the FIND technology, thus proving the benefits of the device, i.e., isolation of rare fungal strains that are challenging to cultivate in the laboratory as crucial growth factors are missing in artificial medium.

For only four species among the 12 isolates (*A. umbrinum, C. globosum, C. rosea, S. brevicaulis)* secondary metabolites were described in the literature. In the case of *C. globosum* these findings were limited to mycotoxins [34] where in the other cases the isolated structures showed remarkable activities in the field of anticancer and antimicrobial agents [35–40].

*H. alpina* is one of only six species known from the less examined genus *Heydenia,* and was mistaken for a member of the genus *Onygena* by Fischer because of its morphological similarities [41–42]. Molecular data now allow to distinguish both genera from each other [43]. Strains of *H. alpina* were isolated from areas with very harsh conditions, e.g., Antarctic lakes and naked silican rock [43–44]. On the basis of its extraordinary growth behaviour, the *H.* cf. *alpina* (strain 824) isolated during this study was selected for further chemical investigations. This led to the isolation of two novel compounds (**1**, **2**). Compounds **1** and **2** belong to the bergamotene type of sesquiterpenoids, for which manifold ecological functions were reported [45]. The biological role of the closely related (+)-(*E*)-*endo*-β-bergamoten-12-oic acid in *Lycopersicon hirsutum* was well investigated. Present in the trichomes of the leaves, it serves as an oviposition stimulant to the moth *Heliothis zea* in LA 1777 [46]. On the other hand, (+)-(*E*)-*endo*-β-bergamoten-12-oic acid showed repellent activity against arthropod mites. In fact, *L. hirsutum* is highly resistant to arthropod herbivores [47]. The bergamotene derivatives clavigerins, first isolated from the liverwort *Lepidolaena clavigera* also showed insect antifeedant activities [48]. Further research is necessary to shed light on the ecological role of **1** and **2**.

## Conclusion

In conclusion the FIND adds a new isolation technique for filamentous fungi to the existing methods. FIND allows high throughput approaches and overcomes limitations in nutrition and growth conditions compared to artificial broths and laboratory cultivation. It enables to obtain axenic fungal cultures in a single isolation experiment and at the same time restrains fast growing fungi in favour of rare and less examined species. FIND ultimately supports the isolation of novel natural products by providing promising specimen from an environment of interest.

## Experimental

### FIND

The device was crafted from polyoxymethylene by the technical staff of the Helmholtz-Institute for Radiation- and Nuclear Physics, Nussallee 14–16, 53115 Bonn. The central plate (127 mm by 92 mm by 1 mm) and the two symmetrical upper and lower plates, the latter with screw threads, contain 96 through-holes 2 mm in diameter arranged in an 8 by 12 grid in a way that it perfectly aligns with the wells of a 96-well plate. The array of through-holes can be covered by one 142 mm polycarbonate membrane filter with a pore size of 0.03 micron (Sterlitech corp., Kent, WA). The device is sealed by six hex bolts size M3.

### General experimental procedures

Optical rotations were measured with a Jasco DIP 140 polarimeter. ECD spectra were taken on a Jasco J-810 CD spectropolarimeter. UV and IR spectra were obtained using Perkin-Elmer Lambda 40 and Perkin-Elmer Spectrum BX FTIR instruments, respectively. All NMR spectra were recorded in MeOH-*d*_4_ using a Bruker Avance 300 DPX spectrometer. Spectra were referenced to residual solvent signals with resonances at δ_H/C_ 3.35/49.0. HRESIMS were recorded on a LTQ Orbitrap mass spectrometer.

HPLC was performed on a Waters HPLC system equipped with a 1525µ binary pump, a 2998 PDA detector, Breeze 2 software and a Rheodyne 7725i injection system. A Macherey-Nagel Nucleoshell C_18_ column (250 mm × 4.6 mm; 5 µm), Nucleodur PolarTec column (250 mm × 4.6 mm; 5 µm), Pyramid C_18_ column (250 mm × 4.6 mm; 5 µm) and Phenomenex Kinetex C_18_ column (250 mm × 4.6 mm; 5 µm) as well as a Phenomenex Aqua C_18_ column (250 mm × 10 mm; 5 µm) were used.

### Taxonomic identification of strains

Sequencing of the obtained isolates from the FIND experiments was performed by P. Massart and C. Decock at the Belgium coordinated collections of microorganisms (BCCM/IHEM, Brussels) by extracting DNA using their usual protocols and genomic DNA extraction kits and generation of partial sequences of DNA loci using standard primers of ITS1, ITS2, ITS4, 5.8S, RPBII, tef1α and β-tubulin. Digitalized and formatted DNA sequences were compared to reference sequences using BLASTn. ID% of the ITS regions was used to identify each strain. If multiple strains came into question the ID% of other gene sequences were taken into consideration to find the best match. Additionally, macromorphology was examined by colony observation using a Leica MZ6 stereomicroscope (Leica, Wetzlar, Germany) and micromorphology was examined with an Olympus BX51 polarizing microscope (Olympus, Shinjuku, Japan) by analysis of conidia and mycelium to support the bioinformatic suggestions.

### Fermentation, extraction and isolation

For cultivation of the marine isolates from the diffusion chamber device experiments inside and outside of the device a minimal nutrient medium – FIND agar medium (FAM: 0.05% casamino acids, 0.025% fish peptone, 1.5% agar in sea water from the given sample) – was compiled to oppress growth of fast-growing fungi like common air moulds. For soil samples fish peptone was exchanged with meat peptone and deionized water was added instead of sea water. To the cooled autoclaved medium 250 mg penicillin G potassium salt (Roth, CN29.1) and streptomycin sulfate (Sigma-Aldrich, S6501-26G) were added via sterile filtration obtaining a concentration of 250 mg/L each to prevent bacterial growth.

For scale-up fermentation, *H*. cf. *alpina* was cultivated in 42 Fernbach flasks containing 150 mL of BMS agar medium (20 g biomalt in 1 L artificial seawater) each at 25 °C for 28 days. The fermented material of every two Fernbach flasks was extracted three times with 300 mL ethyl acetate (EtOAc) and the organic solvent was evaporated under vacuum to yield 2 g of crude extract. This crude extract was dissolved in 100 mL 80% (v/v) aqueous methanol (MeOH) and successively shaken three times with 100 mL petroleum ether in a separation funnel. MeOH solubles (250 mg) were fractionated by vacuum liquid chromatography (VLC) over Polygoprep 60-50 C_18_ stationary phase (Macherey-Nagel) using gradient elution from 0:100 (MeOH/H_2_O) to 100 % MeOH (25% steps) to yield 4 fractions. 100 mL of the mobile phase was used for each fraction. Fraction 3 was again fractionated by VLC under the before mentioned conditions to yield 4 subfractions: 3.1–3.4. Fraction 3.2 (44 mg) was analysed with the NMR system. Complex spectra obtained from VLC fraction 3.2 by NMR analysis indicated the presence of a wide range of secondary metabolites. This VLC fraction was thus subsequently subjected to further chromatographic separation.

RP-HPLC separation of VLC fraction 3.2 (MeOH/H_2_O 55:45 Phenomenex Luna column (250 × 10 mm, 5 µm, 2 mL/min) obtained 10 fractions (3.2H1–3.2H9). Further HPLC of 3.2H7 and 3.2H9 resulted in the isolation of **1** (3 mg) and **2** (4 mg).

**Heydenoic acid A** (**1**): yellow oil; [α]_D_^25^ −50.0 (*c* 0.24, MeOH); UV (MeOH) λ_max_ (log ε): 203 (4.0), 261 (3.5) nm; CD (*c* 4.0 × 10^−3^ M, MeOH) λ_max_ (Δ ε) 203 (1.33), 207 (1.21), 213 (−0.57), 321 (−0.75); IR ν_max_: 3648, 2930, 1706, 1683, 1558, 1540, 1507, 1435, 1376, 1237, 634 cm^−1^; HRESI-TOF-MS *m/z*: [M + H]^+^ calcd for C_15_H_21_O_3_ 249.1481; found, 249.1485. For ^1^H and ^13^C NMR data see Supporting Information File 1, Table S4.

**Heydenoic acid B** (**2**): yellow oil; [α]_D_^25^ −34.9 (*c* 0.33, MeOH); UV (MeOH) λ_max_ (log ε): 203 (3.8), 258 (3.5) nm; CD (*c* 4.0 × 10^−3^ M, MeOH) λ_max_ (Δ ε) 208 (0.57), 218 (0.45), 321 (−0.38); IR ν_max_: 3346, 2927, 1670, 1558, 1540, 1507, 1436, 1376, 1237, 1037, 634 cm^−1^; HRESI-TOF-MS *m/z*: [M + H]^+^ calcd for C_15_H_23_O_3_ 251.1641; found, 251.1642. For ^1^H and ^13^C NMR data see Supporting Information File 1, Table S5.

### Agar diffusion assay

Culture plates (5% sheep blood Columbia agar, BD) were overlaid with 3 mL tryptic soy soft agar, inoculated with TSB (Tryptic soy broth, Oxoid) growth suspension of the bacteria to be tested. Compounds were diluted to a concentration of 1 mg/mL (syringomycin 0.5 mg/mL) with DMSO and 3 µL of this dilution were placed on the surface of the agar. Compounds diffuse into the agar and the size of the inhibition zone was measured after 24 hours of incubation at 37 °C.

### Disk diffusion assay

Antimicrobial tests of isolated compounds were performed by Ekaterina Egereva (Institute for Pharmaceutical Biology, University of Bonn) following the method described by Schulz et al. [49]. The bacteria *Bacillus megaterium* and *Escherichia coli* were used as representatives for Gram-positive and Gram-negative bacteria. *Microbotryum violaceum* (Basidiomycete), *Eurotium rubrum* (Ascomycete), and *Mycotypha microspora* (Zygomycete) were used as fungal test organisms.

Raw extracts were dissolved in MeOH to give a concentration of 1 mg/mL per test sample. 50 µL (equivalent to 50 µg) of each solution were pipetted onto sterile filter disks (diameter: 9 mm, Carl Roth GmbH & Co. KG, KA08.1) which was then placed onto the appropriate agar medium and sprayed with a suspension of the test organism. Growth media, preparation of spraying suspensions and conditions of incubation were carried out according to Schulz et al. [49]. For tested samples, a growth inhibition zone ≥3 mm and/or a complete inhibition ≥1 mm, measured from the edge of the filter disk, were regarded as a positive result. Growth inhibition was defined as follows: growth of the appropriate test organism was significantly inhibited compared to a negative control; total inhibition: no growth at all in the appropriate zone. Benzylpenicillin (1 mg/mL MeOH), streptomycin (1 mg/mL MeOH) and miconazole (0.5 mg/mL DCM) were used as positive controls.

### Cytotoxicity assay

HEK293 cells were seeded into a PDL-coated 96-well plate (25.000/well) and allowed to settle for 2 hours prior to addition of the compounds (solubilized in water). After incubating for 22 hours, CellTiter Blue reagent (Promega) has been added. After another 2 hours of incubation the fluorescence has been detected using FlexStation 3 (Molecular Devices).

### FIND procedure

For experiment 1, 5 mL of soil (Falcon tube) was diluted in 45 mL of sterile-filtered NaCl 0.85% solution (Ringer solution). This solution underwent manual agitation for 10 minutes and ultrasonication using a Bandelin Sonorex at 100% amplitude (35 kHz) two times for 5 seconds to detach cells from sample surfaces. After centrifugation using an Thermo Scientific Heraeus Multifuge X1R at 130 gravities for 5 minutes, cell counting was performed on the supernatant using a Neubauer Improved or Thoma chamber on an Olympus BX51 polarizing microscope at 100× magnification, and the abundance of bacteria was calculated by counting bacterial cells in all 25 medium squares of the central large square or all 16 medium squares of one of the large square, respectively. With Neubauer Improved chambers the supernatant was exchanged three times, for Thoma chambers three different large squares were counted. All the cells within each medium square (totally) together with those that are over the top and right sides of the square (even partially) were considered inside of a square. Mean cell counts were calculated and converted into concentrations by multiplying cell counts with the reciprocal value of the volume of a small square in L, considering possible dilution steps. For experiments 2 and 3, samples were treated alike, but instead of a Ringers solution, sea water from the respective sampling site was used. For experiment 4 only sea water was used.

With the calculated abundances 2 × 10^13^ as well as 7 × 10^9^, 13 × 10^9^ and 8 × 10^9^ bacterial cells/L for experiments 1–4, respectively, volumes between 10 and 50 mL of the solutions were taken according to the corresponding assumed bacteria-to-fungi ratios of the given sample type. These volumes, e.g., 16, 24 and 42 mL, respectively, were then added to a volume of FAM solution to obtain a concentration of 320.000 fungal parts per litre.

For experiment 4, 500 mL portions of a 10 L sample were poured through a sieve with a mesh size of 35 (0.5 mm) onto Whatman filter paper disks 602H with a pore size of 2 µm attached to a Büchner funnel. The funnel was put onto a 2 L suction bottle which was connected to a KNF Neuberger N811 KN.18 vacuum pump that provided negative pressure in the bottle and therefore ensured filtration of the sample portions. The residues on the filter paper disks (5 mL) were resuspended in 5 mL autoclaved sea sample and also mixed with 15 mL warm FAM solution resulting in a concentration of approximately 320 000 fungal parts/L. The prepared FAM solutions containing the corresponding samples were homogenized by stirring and the central plate was dipped into it. The desired target concentration is necessary to allow only one fungal part per through hole in the central plate. As the agar solidifies, the specimen is trapped in the chamber. A semipermeable polycarbonate membrane filter was attached to both sides of the central plate. The upper and lower parts of the FIND were connected to each other with six M3 hex bolt screws, sandwiching the central plate with the attached membranes between them. To ensure the devices seal commercial aquarium safe and antifungal-less silicone (Soudal) was applied to the edges of the device. The silicone was given time to dry and the device was inserted into a tank containing 40 L of soil for experiment 1, 2 L of sea water and sediment for experiment 2, 20 L of sea water and sediment for experiment 3 and sea water for experiment 4. An aquarium powerhead pump provided water current for simulation, oxygenation and distribution purposes for experiments 2–4. Evaporated tank water was replaced by autoclaved deionized water for experiments 2–4 and the soil in experiment 1 was watered with autoclaved deionized water every two days.

After four weeks of incubation inside of the tank the device was removed, rinsed with autoclaved deionized water to remove sediment and soil and opened under a laminar air flow cabinet to transfer each agar plug to a corresponding agar plate for cultivation. For transfer into 96 well plates a Boekel replica plater was used to punch all agar plugs from the central plate into the corresponding wells containing liquid FAM medium. For transfer onto agar plates, autoclaved wooden toothpicks were used to pick each agar plug from the central plate manually and streak them onto agar plates. The plates were stored in a Memmert BE500 incubator at 25 °C for four weeks.

### Salt dependency experiments

Artificial sea water was used for the preparations of the agar plates. The stock solution containing 23.48 g NaCl, 10.61 g MgCl_2_·6H_2_O, 3.92 g Na_2_SO_4_, 1.47 g CaCl_2_·2H_2_O, 0.66 g KCl, 0.19 g NaHCO_3_, 0.1 g KBr, 0.04 g SrCl_2_, 0.03 g H_3_BO_3_ per litre deionized water equals 35‰ salinity. Dilutions with 7, 14, 21 and 28‰ water were achieved by adding deionized water to portions containing 480, 360, 240, 120 mL of the stock solutions, respectively, to obtain 600 mL each. For the 0‰ medium 600 mL deionized water was used. 20 mg biomalt and 15 mg agar per litre were added to the solutions and pH was adjusted at 7 by adding NaOH/HCl. After autoclaving for 20 minutes at 121 °C using an H+P Varioklav autoclave 100 mL of cooled agar solution were poured into petri dishes 130 mm in diameter. Agar plugs (1 cm^2^) cut from pure Petri dish cultures of *Cladosporium allicinum* and *Heydenia* cf. *alpina* were transferred onto the centre of the prepared agar plates. The experiment was performed in triplicate for each salinity level.

## Supporting Information

File 1 Genomic sequences of isolated fungi, data on bioactivity and halotolerance, spectroscopic data of compounds **1** and **2** from *Heydenia* cf. *alpina* strain 824.
