# Supplementary material for: Isolation of fungi using the diffusion chamber device FIND technology
Source: Beilstein J Org Chem. 2019 Sep 19;15:2191–203. doi: 10.3762/bjoc.15.216 (PMC6774082; doi:10.3762/bjoc.15.216)
Supplement: File 1 — Genomic sequences of isolated fungi, data on bioactivity and halotolerance, spectroscopic data of compounds 1 and 2 from Heydenia cf. alpina strain 824. [file Beilstein_J_Org_Chem-15-2191-s001.pdf]

BEILSTEIN JOURNAL OF ORGANIC CHEMISTRY

Supporting Information

for

Isolation of fungi using the diffusion chamber device FIND technology

Benjamin Libor, Henrik Harms, Stefan Kehraus, Ekaterina Egereva, Max Crüsemann and Gabriele M. König

Beilstein J. Org. Chem. **2019,** *15,* 2191–2203. doi:10.3762/bjoc.15.216

Genomic sequences of isolated fungi, data on bioactivity and halotolerance, spectroscopic data of compounds 1 and 2 from *Heydenia* cf. *alpina* strain 824

License and Terms: This is a supporting information file under the terms of the Creative Commons Attribution License (http://creativecommons.org/licenses/by/4.0). Please note that the reuse, redistribution and reproduction in particular requires that the authors and source are credited.
The license is subject to the *Beilstein Journal of Organic Chemistry* terms and conditions: (https://www.beilstein-journals.org/bjoc)

Table S1: Salinity dependency of the growth of *Cladosporium allicinum* on agar plates S2
Table S2: Salinity dependency of the growth of *Heydenia* cf. *alpina* on agar plates.......................... S2
Biological activities S2
Table S3: Agar diffusion assay with extracts of fungal isolates from experiments 1-4 .... S3
Cytotoxicity assay S4
Figure S3: CellTiter-Blue cell viability assay S4
ITS sequences.. S4
Alternaia armoraciae No. 830 .... S4
Auxarthron cf. umbrinum No. 825 .... S5
Cadophora sp. No. 829 .... S5
Chaetomium globosum No. 827 .... S6
Chrysosporium sp. No. 831 .... S6
Cladosporium allicinum No. 823 .... S7
Clonostachys rosea No. 821 .... S7
Heydenia cf. *alpina* No. 824.. S7
Ilyonectria europaea No. 822.. S8
Leucothecium sp. No. 828 .... S8
Metarhizium carneum No. 826.. S8
Scopulariopsis brevicaulis No. 832.. S9
Spectroscopic data of 1 and 2 .... S10
Figure S2: CD spectrum of heydenoic acid A (**1**) in MeOH S10
Figure S3: CD spectrum of heydenoic acid B (**2**) in MeOH S10
Table S4: ^{13}C NMR (75 MHz) and ^{1}H NMR (300 MHz) data of heydenoic acid A (**1**) in MeOD ... S11
Table S5: ^{13}C NMR (75 MHz) and ^{1}H NMR (300 MHz) data of heydenoic acid B (**2**) in MeOD ... S12
1D and 2D NMR spectra of heydenoic acid A (1) S13
Figure S4: ^{1}H NMR of heydenoic acid A (**1**) (300 MHz, in MeOD)................................. S13
Figure S5: ^{13}C NMR of heydenoic acid A (**1**) (75 MHz, in MeOD) S14
Figure S6: ^{1}H,^{1}H-COSY spectrum for heydenoic acid A (**1**) in MeOD S15
Figure S7: HSQC spectrum for heydenoic acid A (**1**) in MeOD.. S16
Figure S8: HMBC spectrum for heydenoic acid A (**1**) in MeOD.. S17
Figure S9: NOESY spectrum for heydenoic acid A (**1**) in MeOD S18
1D and 2D NMR spectra of heydenoic acid B (2) S19
Figure S10: ^{1}H NMR of heydenoic acid B (**2**) (300 MHz, in MeOD) S19
Figure S11: ^{13}C NMR of heydenoic acid B (**2**) (75 MHz, in MeOD) S20
Figure S12: ^{1}H,^{1}H-COSY spectrum for heydenoic acid B (**2**) in MeOD S21
Figure S13: HSQC spectrum for heydenoic acid B (**2**) in MeOD.. S22
Figure S14: HMBC spectrum for heydenoic acid B (**2**) in MeOD.. S23
Figure S15: NOESY spectrum for heydenoic acid B (**2**) in MeOD S24

Table S1: Salinity dependency of the growth of *Cladosporium allicinum* on agar plates

	colony diameter after 14 days [mm]					
Salinity	plate 1	plate 2	plate 3	$\bar{x}$	σ^2	σ
0‰	55	53	54	54	1	±1
7‰	74	71	71	72	2	±1
14‰	90	87	89	89	2	±1
21‰	94	92	89	92	4	±2
28‰	106	106	108	106	1	±1
35‰	107	108	111	109	3	±2

σ = standard deviation

Table S2: Salinity dependency of the growth of *Heydenia* cf. *alpina* on agar plates

	colony diameter after 14 days [mm]					
Salinity	plate 1	plate 2	plate 3	$\bar{x}$	σ^2	σ
0‰	68	66	67	67	1	±1
7‰	123	125	123	124	1	±1
14‰	130	130	130	130	0	±0
21‰	130	130	130	130	0	±0
28‰	130	130	130	130	0	±0
35‰	130	130	130	130	0	±0

σ = standard deviation

Biological activities

Both compounds (**1**,**2**) were tested for antimicrobial activities against *Staphylococcus aureus* 133, *Bacillus subtilis* 168, *Micrococcus luteus* 4698, *Arthrobacter crystallopoietes* DSM 20117, *Escherichia coli* I-11276b, and *Klebsiella pneumoniae* sp. *ozeanae* I-10910 using agar diffusion assays on Culture plates (5% sheep blood Columbia agar, BD) overlayed with growth suspension of the bacteria to be tested. None of the compounds showed antimicrobial effects at concentrations of up to 100 µM.

Both compounds were tested for cytotoxicity in HEK293 cells using CellTiter-Blue cell viability assay. None of the compounds showed cytotoxic effects at concentrations of up to 100 µM.

Table S3: Agar diffusion assay with extracts of fungal isolates from experiments 1–4

	Inhibition zone [mm]				
Extract/chemical 1mg/mL	*Escherichia coli*	*Bacillus megaterium*	*Eurotium rubrum*	*Microbotryum violaceum*	*Mycotypha microspora*
Alternaria armoraciae	n.n.	**2**	n.n.	n.n.	n.n.
Auxarthron umbrinum	n.n.	**1**	n.n.	n.n.	n.n.
Chaetomium globosum	n.n.	**1,5**	n.n.	n.n.	n.n.
Chrysosporium sp.	n.n.	**1**	n.n.	n.n.	n.n.
Cladosporium allicinum	n.n.	**4**	n.n.	n.n.	n.n.
Clonostachys rosea	n.n.	**5**	n.n.	n.n.	n.n.
Cadophora sp.	n.n.	n.n	n.n.	n.n.	n.n.
Heydenia cf. alpina	n.n.	**5**	n.n.	n.n.	n.n.
Ilyonectria europaea	n.n.	**3**	n.n.	**3**	n.n.
Leucothecium sp.	n.n.	n.n	n.n.	n.n.	n.n.
Metarhizium carneum	n.n.	n.n	n.n.	n.n.	n.n.
Scopulariopsis brevicaulis	n.n.	n.n	n.n.	n.n.	n.n.
Benzylpenicillin	6	n.n.	n.n.	n.n.	n.n.
Streptomycin	n.n.	10	n.n.	n.n.	n.n.
Miconazole	n.n.	n.n	10	10	15

n.n. = no inhibition

Cytotoxicity assay

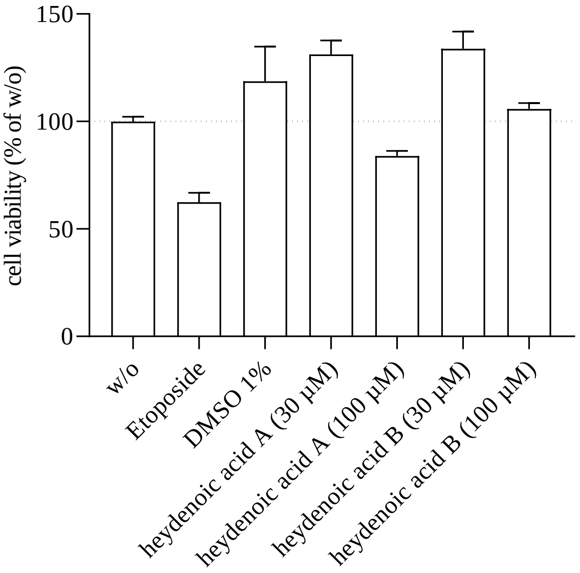

Figure S1: CellTiter-Blue cell viability assay

ITS sequences

***Alternaia armoraciae* No. 830**
>Z1828-4418AI

TCCGTAGGTGAACCTGCGGAGGGATCATTACACAATATGAAGGCGGGCTGGATCCCTCCTGC
TGGGCACTGCTTCACGGCGTGCGCAGTAGGGGCCGGCCCTGCTGAACTATTCACCCGTGTCTT
TTGCGTACCTCTTGTTTCCTGGGCGGGCTCGCCCGCCACCAGGACCAACCATAAACCTTTTTG
TAATAGCAATCCGCGTCAGTAACAACGTAATTAATTACAACTTTCAACAACGGATCTCTTGGT
TCTGGCATCGATGAAGAACGCAGCGAAATGCGATACGTAGTGTGAATTGCAGAATTCAGTGA
ATCATCGAATCTTTGAACGCACATTGCGCCCTTTGGTATTCCAAAGGGCATGCCTGTTCGAGC
GTCATTTGTACCCTCAAGCTTTGCTTGGTGTTGGGCGTCTTTGTCTCCAGCTTGCTGGAGACTC
GCCTTAAAGTCATTGGCAGCCGGCCTACTGGTTTCGGAGCGCAGCACAAGTCGCGCTCTTCA
TCCAGCCAAGGTCAGCGTCCAGCAAGCCTTTTTTCAACCTTTGACCTCGGATCAGGTAGGGAT
ACCCGCTGAACTTAAGC

>Z1828-4418AEFCL

AGAAGGTACGACATCACTCTTTCTTCCCACATGGGCTGTCGCAACCACCCGGTGCATCTCTGA
GCGCGCCGCCATTTCTGGCTTATCGCGATGAGGGGCATTTTTGGGTGGTGGGGTTGTGCGAA
CTTTTACGCGCTAGCGCTAGTCTGCATGCGGCCTTCGCGAGCCCCAACACCATGACGCACATC
CAATTTCCCCAATTCGGCCACAGCTAACAAGCCTCACAGGAAGCCGCCGAACTCGGTAAGG

>Z1828-4418BI

TCCGTAGGTGAACCTGCGGAGGGATCATTACACAATATGAAGGCGGGCTGGATCCCTCCTGC
TGGGCACTGCTTCACGGCGTGCGCAGTAGGGGCCGGCCCTGCTGAACTATTCACCCGTGTCTT
TTGCGTACCTCTTGTTTCCTGGGCGGGCTCGCCCGCCACCAGGACCAACCATAAACCTTTTTG
TAATAGCAATCCGCGTCAGTAACAACGTAATTAATTACAACTTTCAACAACGGATCTCTTGGT
TCTGGCATCGATGAAGAACGCAGCGAAATGCGATACGTAGTGTGAATTGCAGAATTCAGTGA
ATCATCGAATCTTTGAACGCACATTGCGCCCTTTGGTATTCCAAAGGGCATGCCTGTTCGAGC
GTCATTTGTACCCTCAAGCTTTGCTTGGTGTTGGGCGTCTTTGTCTCCAGCTTGCTGGAGACTC
GCCTTAAAGTCATTGGCAGCCGGCCTACTGGTTTCGGAGCGCAGCACAAGTCGCGCTCTTCA
TCCAGCCAAGGTCAGCGTCCAGCAAGCCTTTTTTCAACCTTTGACCTCGGATCAGGTAGGGAT
ACCCGCTGAACTTAAGC

***Auxarthron cf. umbrinum* No. 825**
>Z1828-4420I

TCCGTAGGTGAACCTGCGGAAGGATCATTAAAGCGTCGAGCCTGCGCCTCCCGGCGTAGGTG
AAACCCCACCCGTGACTACTACACCACATGTTGCTTTGGCGGGCCCGCCTCTGGCTGCCGGG
GTTTCTCTGGATAGCGCCCGCCAAAGATACACTGAACTTCTGTGAAACTGGATGTCTGAGTTG
ATATCAATCATTAAAACTTTCAACAATGGATCTCTTGGTTCCGGCATCGATGAAGAACGCAG
CGAAATGCGATAAGTAATGTGAATTGCAGAATTCCGTGAATCATCGAATCTTTGAACGCACA
TTGCGCCCTCTGGTATTCCGGAGGGCATGCCTGTCCGAGCGTCATTGCAACCTTCAAGCGCGG
CTTGTGTGTTGGGCCTCGTCCCCCGTGGACGGGCCTCAAAGGCAGTGGCGGCGTCCGTTTTGG
TGCCCGAGCGTATGGGAATTCTATACCGCTTCAAGGCCCGGCGGCGCTGGTCAAGACCAATT
TTTATCGGTTGACCTCGGATCAGGTAGGGATACCCGCTGAACTTAAGCATA

***Cadophora* sp. No. 829**
>Z1876-4467I

TCCGTAGGTGAACCTGCGGAAGGATCATTAATAGAGTAAGGGCGAAGCTGTAAAAGGCCGA
GCTCTGACCTCCACCCTTGAATAAACTACCTTCGTTGCTTTGGCGGGTCGCCTCGTGCCAGCG
GCTTCGGCTGTTGAGTACCCGCCAGAGGACCACAACTCTTGTTTTTAGTGATGTCTGAGTACT
ATATAATAGTTAAAACTTTCAACAACGGATCTCTTGGTTCTGGCATCGATGAAGAACGCAGC
GAAATGCGATAAGTAATGTGAATTGCAGAATTCAGTGAATCATCGAATCTTTGAACGCACAT
TGCGCCCTCTGGTATTCCGGGGGGCATGCCTGTTCGAGCGTCATTATAACCACTCAAGCTCTC
GCTTGGTATTGGGGTTCGCGTCTTCGCGGCCTCTAAAATCAGTGGCGGTGCCTGTCGGCTCTA
CGCGTAGTAATACTCCTCGCGATTGAGTTCCGGTAGGTTTACTTGCCAACAACCCCCAATCTT
TTAAGGTTGACCTCGGATCAGGTAGGGATACCCGCTGAACTTAAGCATATCAT

>Z1876-4467VS

GTAACGGCGAGTGAAGCGGTAACAGCTCAAATTTGAAAGCTAGCTCTTTTAGGGTTCGCATT
GTAATTTGTAGAAGATGCTTCGGGTGTGGCCCCGGTCTAAGTTCCTTGGAACAGGACGTCAT
AGAGGGTGAGAATCCCGTATGTGACTGGGTGCTTTCGCTCATGTGAAGCTCTTTCGACGAGT
CGAGTTGTTTGGGAATGCAGCTCAAAATGGGTGGTAAATTTCATCTAAAGCTAAATATTGGC
CAGAGACCGATAGCGCACAAGTAGAGTGATCGAAAGATGAAAAGCACTTTGGAAAGAGAGT
TAAACAGTACGTGAAATTGTTGAAAGGGAAGCGCTTGCAACCAGACTTGCGCGTAGTTGATC
ATCCGAGCTTCTGTTTGGTGCACTCTGCTACGCTCAGGCCAGCATCGGTTTCGGTGGTGGGAT
AAAGGCCTTGGGAATGTAGCTCCTCTCGGGGAGTGTTATAGCCCTCGGTGCAATGCCGCCTA

TCGGGACCGAGGACCGCGCTTCGGCTAGGATGCTGGCGTAATGGTTGTAAGCGACCCGTCTT
GAAACACGGACCAAGGAGTCTAACATCTATGCGAGTGTTTGGGTGTCAAACCCATACGCGTA
ATGAAAGTGAACGGAGGTGAGAACCCTTAAGGGTGCATCATCGACCGATCCTGATGTCTTCG
GATGGATTTGAGTAAGAGCATAGCTGTTGGGACCCGAAAGATGGTGAACTATGCGTGAATAG
GGTGAAGCCAGAGGAAACTCTGGTGGAGGCTCGCAGCGGTTCTGACGTGCAAATCGATCGTC
AAATTTGCGTATAGGGGCGAAAGACTAATCGAACCATCTAGTAGCTGGTTCCTGCCGAAGTT
TCCCTCAGGATAGCAGTGTTGAATTCAGTTTTATGAGGTAAAGCGAATGATTAGAGGCCTTG
GGGTTGAAACAACCTTAACCTATTCTCAAACTTTAAATATGTAAGAAGTCCTTGTTACTTAAT
TGAACG

***Chaetomium globosum* No. 827**
>Z1828-4417BT

GGTGCTGCTTTCTGGTACGTCCCAGCAAAGCAAACACTCTTGGCTGATGACAATCGAGACTG
ACTTCTTTTCAGGCAGACCATCTCTGGCGAGCACGGCCTCGACAGCAATGGCGTGTATGTGG
GCATGACAGTTCCCAACCGATAAATCCCCGCTCACCGCTTCGATAGGTACAACGGCACCTCC
GAGCTCCAGCTCGAGCGTATGAACGTGTACTTCAACGAGGTCAGTCGGGTCAAATAATTTTA
CACGACCGAGTGATGGCGTGCTCACAGTATTATACAGGCTTCCGGCAACAAGTATGTTCCTC
GCGCTGTCCTCGTCGACTTGGAGCCCGGCACCATGGATGCCGTCCGTGCCGGCCCCTTCGGCC
AGCTCTTCCGCCCGGACAACTTCGTCTTCGGCCAGTCGGGTGCCGGCAACAACTGGGCCAAG
GGTC

>Z1828-4417RPB2

GCTGAGACCCCGAAGGCCAGGCTGCGGTCTCGTCAAAAACTTGTCACTGATGTGCTACGTCA
GTGTGGGCACCCCGCGGACCCGATCGTTGAGTTCATGATCGCCCGAGGTATGGAAGTGCTCG
AGGAGTACGAACCACTCCGATATCCGAACGCCACCAAGGTGTTCGTAAACGGCACTTGGGTG
GGTGTTCACCAAGACCCAAAGCATCTTGTCACGCTGGTTCAGGGGCTGCGGAGAAAGAACGT
TATCTCGTTTGAGGTTTCGCTCGTTAGAGACATCCGCGACCGTGAGTTCAAGATCTTTTCAGA
TGCGGGTCGGGTGATGAGGCCGCTGTTCACGGTGGAGCAAGAACCGAATGGCGAGAGCGGC
GCTGAGATGGGCGCACTGATCCTGAATAAGGATCATATTGGGCGCCTGAAGATGGACGCAG
AGCTGGGCAAATACCACCCGGACTACTGGGGCTGGCAAGGCCTGTTGAAGTCGGGCGCTATT
GAGTATCTTGATGCTGAGGAGGAGGAGACGGTCATGATCTGCATGACCCCCCAGGATCTTGA
TCAGTTCCGTGCCCGCAAGATGGGAAGGATCGAGCCGGACAACTCCGGGTTGGGCAATAACC
GGATCAAGACGAAACCAAATCCGACAACTCACATGTACACGCACTGCGAGATCCATCCGAG
CATGCTCCTCGGCATCTGCGCAAGCATCATCCCCTTTCCTGATCATAACCAATCGCCCAGGAA
CACATACCAGTCCGCTAT

***Chrysosporium sp.* No. 831**
>Z1876-4466I

TCCGTAGGTGAACCTGCGGAAGGATCATTACAGTGTCTGGAGGCCGACCGGCGGCGTTTCCC
TCACGGGGAGCGTCGTGGCTCGTGCCCCCCCCCACACGTGTTTACTACACCCTGTTGCCTTGG
TGGGTCTGCCCTTGTGGCTGCCGGGGGTCACCGCGTGTGCCCCGGGCCCGTACCCACCGATG
GACACCCTGAACTCTTTATGAATATAGTGTTGTCTGAGCGTTTAGCAAATTAAACAAAACTTT
CAACAATGGATCTCTTGGTTCTGGCATCGATGAAGAACGCAGCGAAATGCGATAAGTAATGT
GAATTGCAGAATTCCGTGAATCATCGAATCTTTGAACGCACATTGCGCCCTCTGGTATTCCGG

GGGGCATGCCTGTTCGAGCGTCATTGCAACCCCTCAAGCACAGCTTGTGTGTTGGGCTCTCGT
CCCCTGTGGACGGGCCTGAAATGCAGTGGCAGCACCGAGATACCGGTGTCTGAGTGTATGGG
CATTGATCATCACTCGATAGACCCGATCGGCGCTGACCGTCAAACCACGATTCCTCGGAATC
ATCCAGTTTTGACCTCGGATCAGGTAGG

***Cladosporium allicinum* No. 823**
>Z1378-2864EF

TCATCGAGAAGTTCGAGAAGGTGAGCACTCTTCCGGCGCCTTGTTGTCTGGTCATCGATGCA
ATCTCTTCGCCCCACCAAGCCACCCCGCCTCGTCGCAATCTGCGATAAGGTGTCTGACGCCTG
GCTTGGCGAAGAACTTGTCTCTCGAGGGACAGACAGCACGCCACCCACTCCTACCTGAACAC
ATCACTGACAATTCACCACAGGAAGCCGCCGAGCTCGGTAAGGGTCCTTCAAGTAA

>Z1378-2864ITS

TTACAAGAACGCCCGGGCTTCGGCCTGGTTATTCATAACCCTTTGTTGTCCGACTCTGTTGCC
TCCGGGGCGACCCTGCCTTCGGGCGGGGGCTCCGGGTGGACACTTCAAACTCTTGCGTAACT
TTGCAGTCTGAGTAAACTTAATTAATAAATTAAAACTTTTAACAACGGATCTCTTGGTTCTGG
CATCGATGAAGAACGCAGCGAAATGCGATAAGTAATGTGAATTGCAGAATTCAGTGAATCAT
CGAATCTTTGAACGCACATTGCGCCCCCTGGTATTCCGGGGGGCATGCCTGTTCGAGCGTCAT
TTCACCACTCAAGCCTCGCTTGGTATTGGGCAACGCGGTCCGCCGCGTGCCTCAAATCGTCCG
GCTGGGTCTTCTGTCCCCTAAGCGTTGTGGAAACTATTCGCTAAAGGGTGTTCGGGAGGCTAC
GCCGTAAAACAACCCCATTTCTAAGGTTGACCTCGGATCAGGTAGGGATACCCGCTGAACTT
AAGCATATCATA

***Clonostachys rosea* No. 821**
>Z2328-2172 ITS

TCCGTTGGTGAACCAGCGGAGGGATCATTACCGAGTTTACAACTCCCAAACCCATGTGAACA
TACCTACTGTTGCTTCGGCGGGATTGCCCCGGGCGCCTCGTGTGCCCCGGATCAGGCGCCCGC
CTAGGAAACTTAATTCTTGTTTTATTTTGGAATCTTCTGAGTAGTTTTTACAAATAAATAAAA
ACTTTCAACAACGGATCTCTTGGTTCTGGCATCGATGAAGAACGCAGCGAAATGCGATAAGT
AATGTGAATTGCAGAATTCAGTGAATCATCGAATCTTTGAACGCACATTGCGCCCGCCAGTA
TTCTGGCGGGCATGCCTGTCTGAGCGTCATTTCAACCCTCATGCCCCTAGGGCGTGGTGTTGG
GGATCGGCCAAAGCCCGCGAGGGACGGCCGGCCCCTAAATCTAGTGGCGGACCCGTCGTGG
CCTCCTCTGCGAAGTAGTGATATTCCGCATCGGAGAGCGACGAGCCCCCTGCCGTTAAACCC
CCCAACTTTTCCAAGGT

***Heydenia* cf. *alpina* No. 824**
>Z2259-3333ITS

TCCGTAGGTGAACCTGCGGAAGGATCATTAAAAAATATAGAATTAATCTTCTGTAAACCCAA
TCTGCGTATTTCTACCTGTTGCTTTCGTGAGACTGTGAACGCAAGTTCCCTCTGGCGCTGTTTT
TAGGAACAGCTGTTGGGGAGTGCTCACGGGAGGTAATTATAAACTCTGTTTTTTTTGAATTTT
GTCTGAATATTGTTTATACATAAACTTTAAAACTTTCAACAACGGATCTCTTGGTTCTCGCAT
CGATGAAGAACGCAGCGAAATGCGATAAGTAGTGTGAATTGCAGAATTCAGTGAATCATCG
AATCTTTGAACGCACATTGCGCCTCCTGGTATTCCGGGAGGCATGCCTGTTCGAGCGTCATTA

AAATCACTCAAGCTTAGGTTTACCTATTGCTTGGTCTTGGAGATGGAAGCCAATTTATTGGAA
TCCTCTTCGAAATTCAATGGCGAAGACCCTTGCTCTCCCAAGTGTAGTAATAACTTATGTCAC
TGAAGGAAGCGAGAAATCTTCTGCCGTAACCCCCATATTTCTATGATTGACCTCGGATCAG
GTAGGGATACCCGCTGAACTTAAGCAT

***Ilyonectria europaea* No. 822**
>Z2328-2173BL ITS

TCCGTTGGTGAACCAGCGGAGGGATCATTACCGAGTTTACAACTCCCAAACCCCTGTGAACA
TACCATATTGTTGCCTCGGCGGTGCCTGTTTCGGCAGCCCGCCAGAGGACCCAAACCCTAGA
TTACATTAAAGTATCTTCTGAGTCAATGATTAAATCAATCAAAACTTTCAACAACGGATCTCT
TGGTTCTGGCATCGATGAAGAACGCAGCGAAATGCGATAAGTAATGTGAATTGCAGAATTCA
GTGAATCATCGAATCTTTGAACGCACATTGCGCCCGCCAGTATTCTGGCGGGCATGCCTGTCC
GAGCGTCATTTCAACCCTCAAGCCCCCGGGCTTGGTGTTGGAGATCGGCGAGCCCTCCGGGG
CGCGCCGTCTCCCAAATATAGTGGCGGTCCCGCTGTAGCTTCCTCTGCGTAGTAGCACACCTC
GCACTGGGAAACAGCGTGGCCACGCCGTGAAACCCCCCACTTCTGAAAGGTTGACCTCGGAT
CAGGTAGGAATACCCGCTGAACTTAAGCATAT

***Leucothecium* sp. No. 828**
>Z1876-4468I

TCCGTAGGTGAACCTGCGGAAGGATCATTATCGAGCCGCCGACGCGGGCCCGCGGTACTTCG
GTGCCGTTGGGTCCCGTAGGCAAATGGCCCAACCCTTGCTTCTTGACAACCATTGTCTCGGCG
GTACCGCGCCTTTCGGGGCCAGCTGGATTCATTCCGGCTTGTGTCCGCCAGAGAACCATTAA
AAATCGTTTATCAGATCGTCTAAGAATGAAATAATTCAATAAAACTTTCAACAACGGATCTC
TTGGTTCCGGCATCGATGAAGAACGCAGCGAAATGCGATAACTAATGTGAATTGCAGAATTC
CGTGAATCATCGAGTCTTTGAACGCACATTGCACCCTCTGGTATTCCGGGGGGTATATCTGTC
CGAGCGTCATTACAACCTTAAAGCACGGCTTTTTATTGGATTCTAGTTCTGCTTCGGCGGGAC
AGGTCCGAAATGGATTAATGACGTCGCGATTACCACGGAATCGAGCGAATGGAATCATTAAC
GCTCTGATTTGAAGTGGCCGACGGTCTTCTGAAGCGGTCCTTTTGGATCGTCTTTTTAACGGT
TGACCTCGG

***Metarhizium carneum* No. 826**
>Z1876-4469I

TCCGTTGGTGAACCAGCGGAGGGATCATTACCGAGTTTACAACTCCCAAACCCCCTGTGAAC
TTATACCATTTACTGTTGCTTCGGCGGGTCATGGCCCCGGGGAAGGACAGCGGTCGCCGTCA
GGCCTCAGCTGCCCGCCCCCGGAAACAGGCGCCCGCCGGGGAACTCAAACTCTTCTGTATTT
CTTTATCTAATATATACTGTCTGAGTAAAAACTAAAATGAATCAAAACTTTCAACAACGGAT
CTCTTGGTTCTGGCATCGATGAAGAACGCAGCGAAATGCGATAAGTAATGTGAATTGCAGAA
TTCAGTGAATCATCGAATCTTTGAACGCACATTGCGCCCGCCAGTATTCTGGCGGGCATGCCT
GTTCGAGCGTCATTTCAACCCTCAAGTCCCCTGTGGACTCGGTGTTGGGGACCGGCGAGACA
GCCGCGGATCTTCTTCCGCAGCGAGTCGCCGCCCCCCAAATGACTTGGCGGCCTCGTCGCGG
CCCTCCTCTGCGTAGTATAGCACACCTCGCAACAGGAGCCCGGCGAATGGCCACTGCCGTAA
AACCCCCCAACTTTTTCAGAGTTGACCTCGAATCAGGTAGGAATACCCGCTGAACTTAAGCA
TATA

***Scopulariopsis brevicaulis* No. 832**

>Z1828-4419I

TCCGTTGGTGAACCAGCGGAGGGATCATTACCGAAGTTACTCTTCAAAACCCATTGTGAACC
TTACCTCTTGCCGCGCGTTGCCTCGGCGGGGAGGCGGGGTCTGGGTCGGCGCGCCCCTCACC
GGGCCGCCGTCCCCGTCCCCGTCCCCGCCGGCCGCGCCAAACTCTAAATTTGAAAAAGCGTA
CTGCACGTTCTGATTCAAAACAAAAAACAAGTCAAAACTTTTAACAACGGATCTCTTGGTTCT
GGCATCGATGAAGAACGCAGCGAAATGCGATAAGTAATGTGAATTGCAGAATTCAGTGAAT
CATCGAATCTTTGAACGCACATTGCGCCCGGCAGCAATCTGCCGGGCATGCCTGTCCGAGCG
TCATTTCTTCCCTCGAGCGCGGCTAGCCCTACGGGGCCTGCCGTCGCCCGGTGTTGGGGCTCT
ACGGGTGGGGCTCGTCCCCCCCGCAGTCCCCGAAATGTAGTGGCGGTCCAGCCGCGGCGCCC
CCTGCGTAGTAGATCCTACATCTCGCATCGGGTCCCGGCGAAGGCCAGCCGTCGAACCTTTT
ATTTCATGGTTTGACCTCGGATCAGGTAGGGTTACCCGCTGAACTTAAGC

>Z1828-4419EF2

GCGCCATTCTCATCATTGCTGCCGGTACTGGTGAGTTCGAGGCTGGTATCTCCAAGGATGGCC
AGACTCGTGAGCACGCTCTCCTCGCCTTCACCCTCGGTGTCAAGAACCTCATTGTCGCCATCA
ACAAGATGGACACTGCCAAGTGGTCCGAGGACCGCTACAGGGAGATCATCAAGGAGACCTC
CAACTTCATCAAGAAGGTCGGCTACAACCCTAAGGCTGTTGCCTTCGTCCCCATCACTGGTTT
CCACGGCGACAACATGATCCACGCCTCCACCAACTGCCCCTGGTACAAGGGCTGGGAGCGTG
AGGTCAAGTCGGGCAAGCTCACCGGCAAGACCCTCCTCGAGGCCATCGACTCCATCGAGCCC
CCCAAGCGTCCTACCGAGAAGCCCCTCCGTCTTCCCCTCCAGGATGTCTACAAGATCGGTGGT
ATTGGCACGGTGCCCGTCGGCCGTATTGAGACCGGTGTCATCAAGCCCGGCATGGTCGTCAC
CTTCGCCCCCTCCAACGTCACCACTGAGGTCAAGTCCGTCGAGATGCACCACGAGCAGCTTC
CCGAGGGTGTCCCCGGTGACAACGTTGGTTTCAACGTGAAGAACGTCTCCGTCAAGGACATT
CGCCGTGGTAACGTTGCCGGTGACTCCAAGAACGACCCCCCTATGGGCGCCGCGTCGTTCCA
GGCTCAGGTCATCGTCCTCAACCACCCTGGTCAGATCGGCGCCGGCTACGCGCCCGTTCTTGA
CTGCCACACTGCCCACATTGCTTGCAAGTTCTCCGAGCTCCTTGAGAAGATCGACCGCCGTAC
CGGTAAGTCGGTTGAGAACAACCCCAAGTTCGTCAAGTCGGGTGACGCTGCCATCGTCAAGA
TGGTTCCCTCCAAGCCCATGTGCGTTGAGGCCTTCACCGAGTACCCCC

Spectroscopic data of compounds 1 and 2

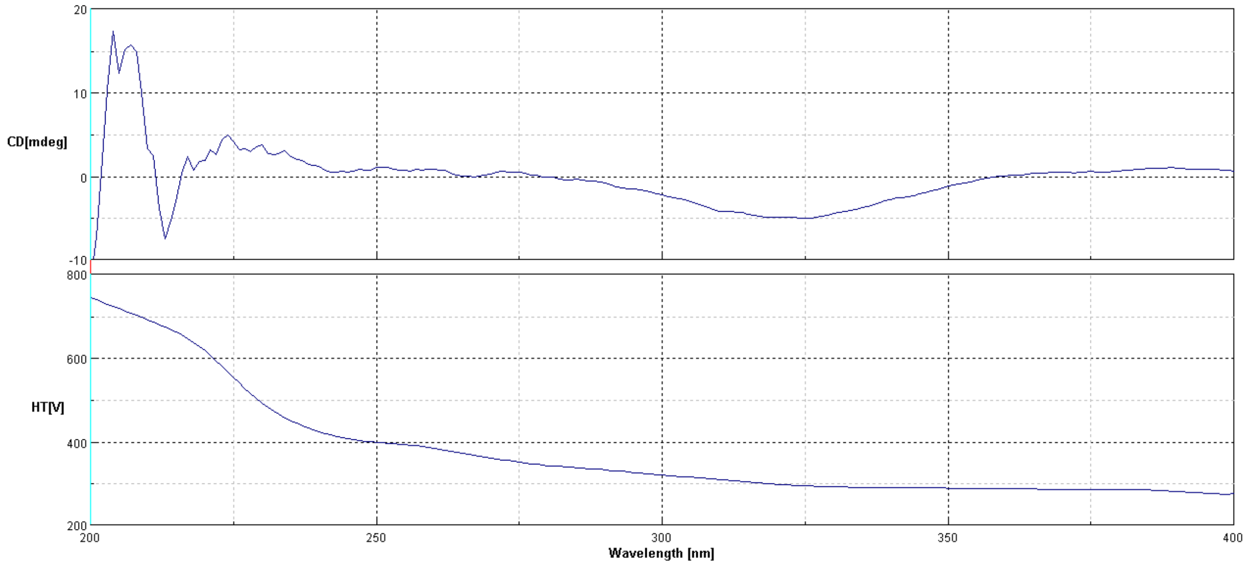

Figure S2: CD spectrum of heydenoic acid A (**1**) in MeOH

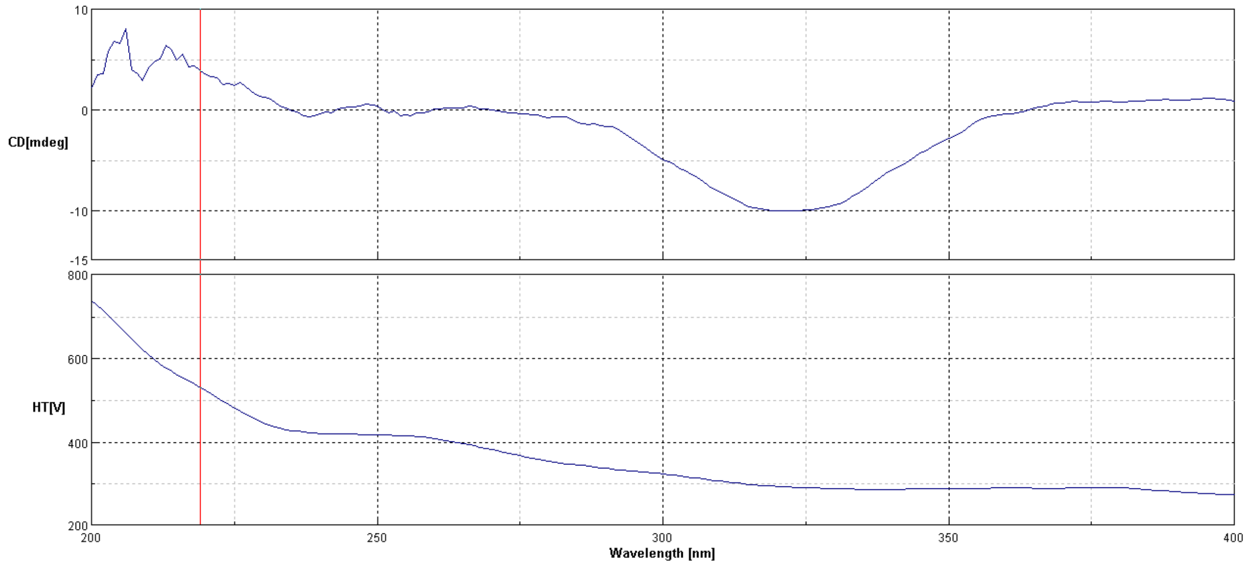

Figure S3: CD spectrum of heydenoic acid B (**2**) in MeOH

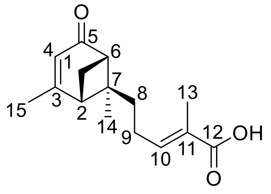

Table S4: ^{13}C NMR (75 MHz) and ^{1}H NMR (300 MHz) data of heydenoic acid A (**1**) in MeOD

C	δ_H	δ_C	COSY	HMBC	NOE
1	a: 2.12, m	41.9, CH_2	1b	2, 3, 5, 6, 7, 14	1b
	b: 2.93, dt (10.0, 6.0)		1a, 2, 6	2, 3, 5, 6, 7	1a, 2, 6, 8
2	2.65, t (6.0)	49.8, CH	1b, 4, 6	3, 4, 6, 7, 15	1b, 8, 14, 15
3		174.1, qC			
4	5.78, q (1.3)	122.1, CH	2, 6, 15	2, 6, 15	15
5		206.6, qC			
6	2.73, t (6.0)	57.2, CH	1b, 4	2, 4, 5, 7	1b, 8, 14
7		58.6, qC			
8	2.07, m	38.2, CH_2	9	7, 9, 14	1b, 6, 14
9	2.32, m	25.0, CH_2	8, 10	7, 8, 10, 11	13, 14
10	6.86, t (7.6)	143.0, CH	9, 13	12, 13	8, 9
11		129.5, qC			
12		177.8, qC			
13	1.89, s	12.5, CH_3	10	10, 11, 12	9
14	1.07, s	19.2, CH_3		2, 6, 7, 8	8, 15
15	2.10, d (1.3)	23.6, CH_3	4	2, 3, 4	2, 4, 14

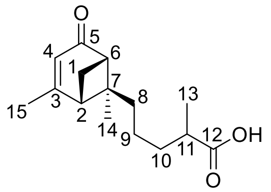

2

Table S5: ^{13}C NMR (75 MHz) and ^{1}H NMR (300 MHz) data of heydenoic acid B (**2**) in MeOD

C	δ_H, (J in Hz)	δ_C, mult	COSY	HMBC	NOE
1	a: 2.10, m	41.9, CH_2	1b	2, 3, 5, 6, 7, 14	1b
	b: 2.89, dt (9.0, 5.6)		1a, 2, 6	2, 3, 5, 6, 7	1a, 2, 6, 8
2	2.59, t (5.6)	49.7, CH	1b, 4, 6	1, 3, 4, 6, 7, 8, 15	1b, 8, 14, 15
3		174.2, qC			
4	5.77, q (1.3)	122.0, CH	2, 6, 15	2, 6, 15	15
5		206.9, qC			
6	2.67, t (5.6)	57.3, CH	1b, 2, 4	1, 2, 4, 5, 7, 8	1b, 8, 14
7		58.8, qC			
8	1.94, m	39.4, CH_2	9	2, 6, 7, 9, 10, 14	1b, 2, 6, 14
9	1.37, m	23.4, CH_2	8, 10	7, 8, 10	
10	a: 1.49, m	35.5, CH_2	9, 10b, 11	9, 12	10b, 13
	b: 1.73, m		9, 10a, 11	9, 11, 13	10a
11	2.50, m	40.7, CH	10a, 10b, 13	9, 10, 12, 13	10a, 13
12		180.8, qC			
13	1.21, d (7.0)	17.8, CH_3	11	10, 11, 12	10a, 11
14	1.01, s	19.3, CH_3		2, 6, 7, 8	8
15	2.09, d (1.3)	23.6, CH_3	4	2, 3, 4	2, 4, 14

1D and 2D NMR spectra of heydenoic acid A (1)

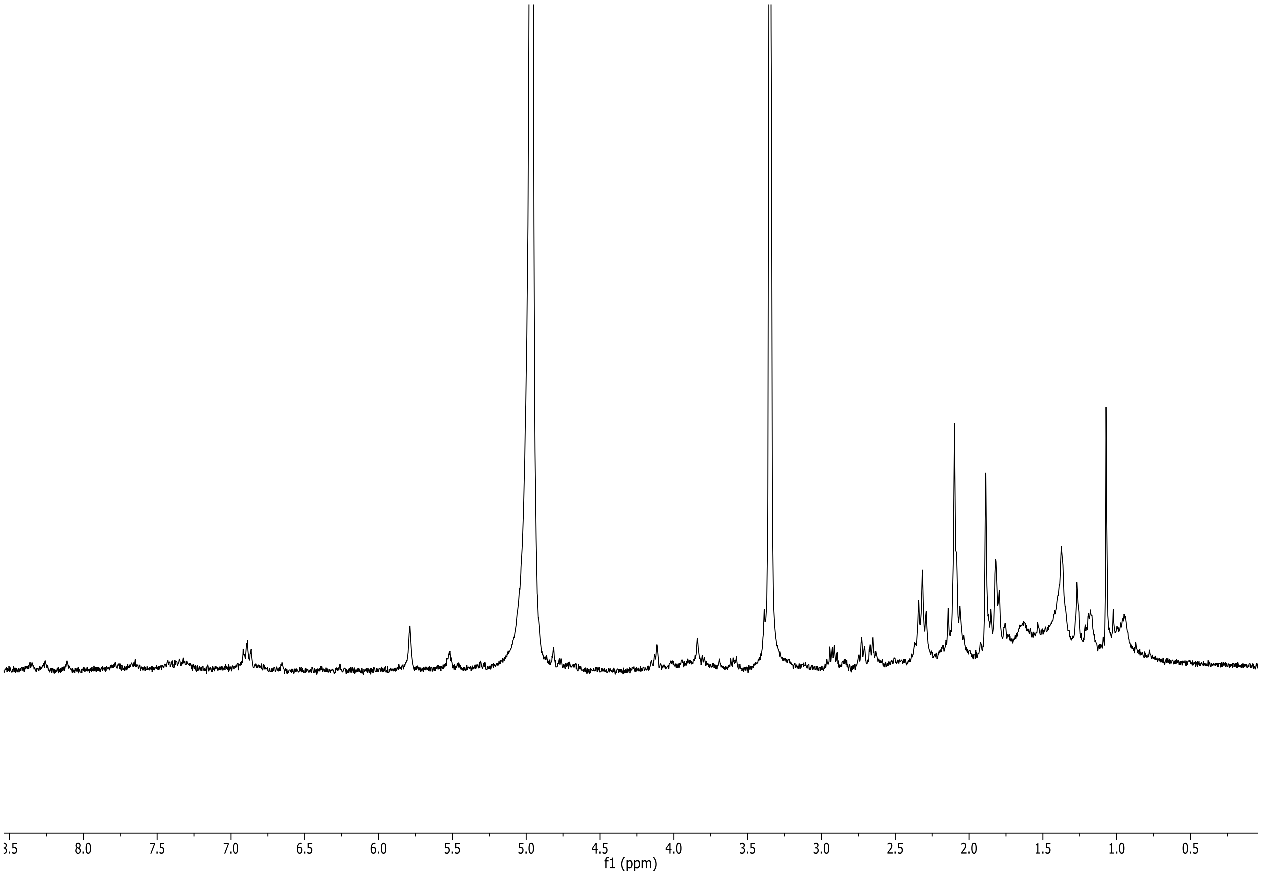

Figure S4: ^{1}H NMR of heydenoic acid A (1) (300 MHz, in MeOD)

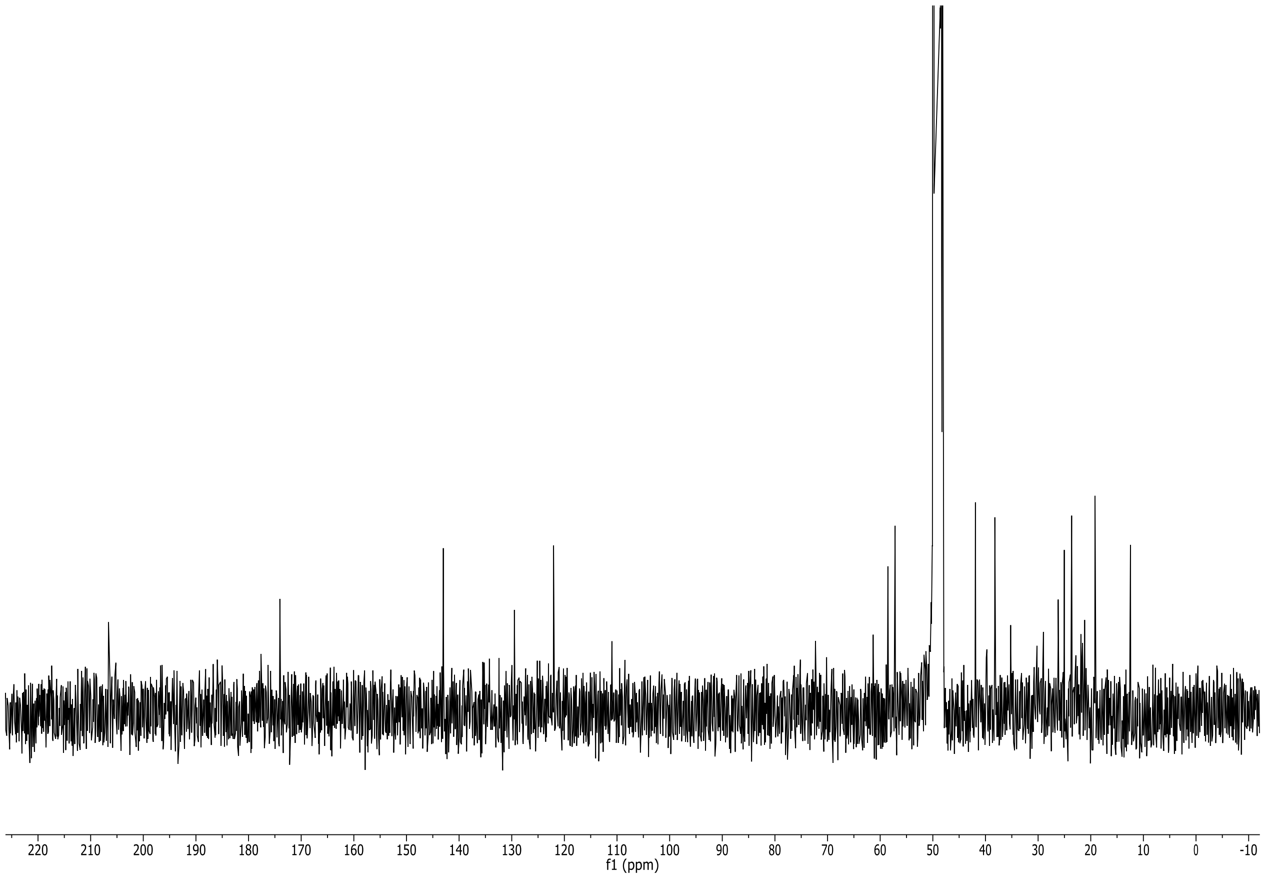

Figure S5: ^{13}C NMR of heydenoic acid A (**1**) (75 MHz, in MeOD)

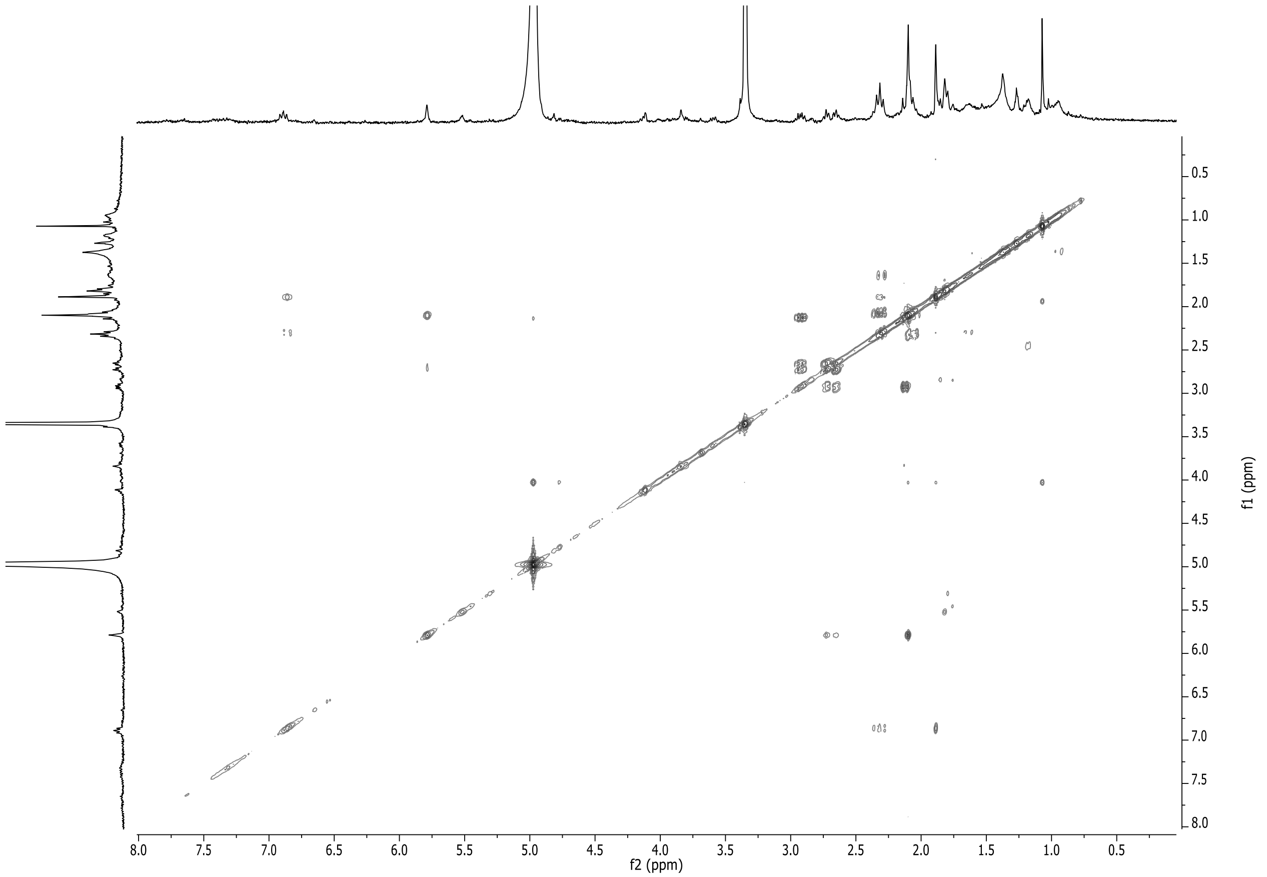

Figure S6: ^{1}H,^{1}H-COSY spectrum for heydenoic acid A (**1**) in MeOD

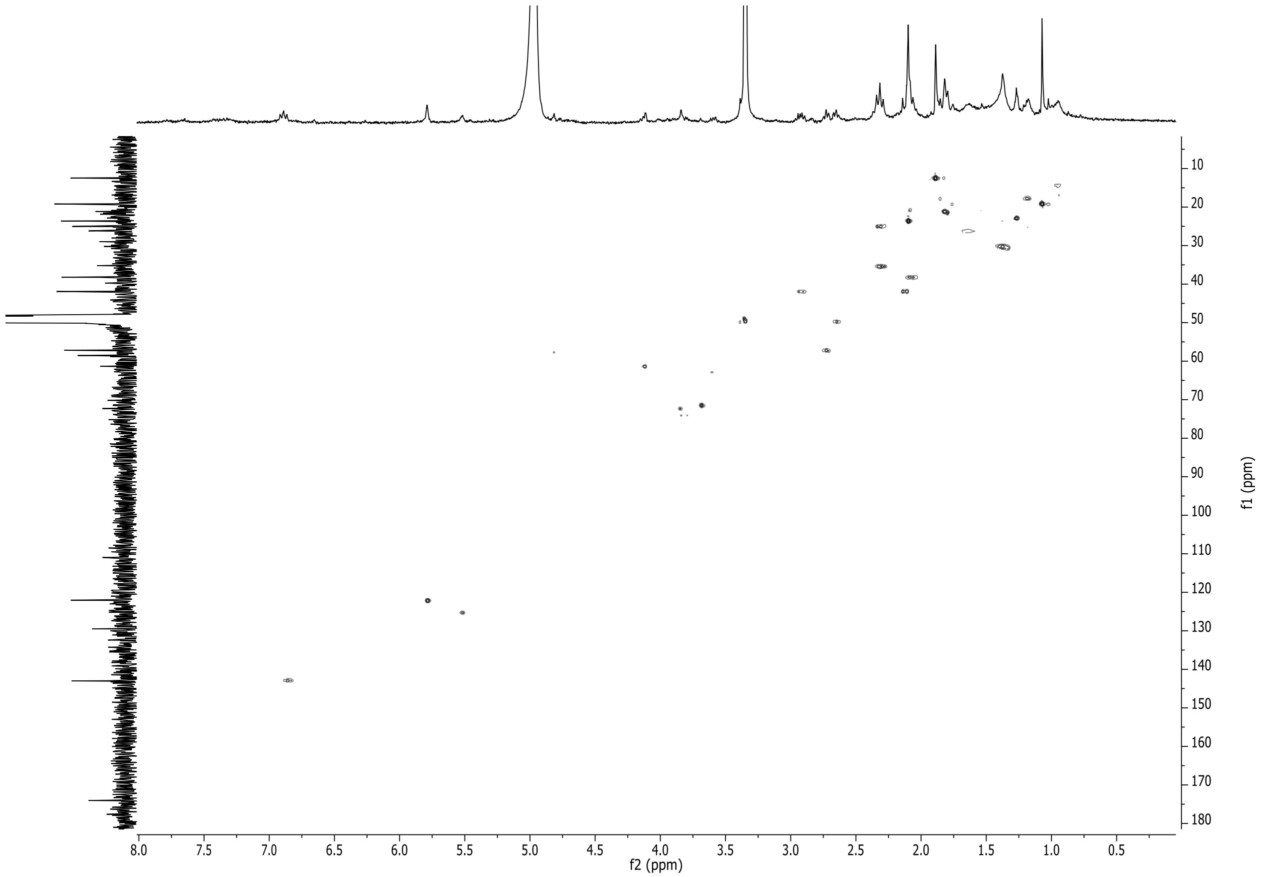

Figure S7: HSQC spectrum for heydenoic acid A (**1**) in MeOD

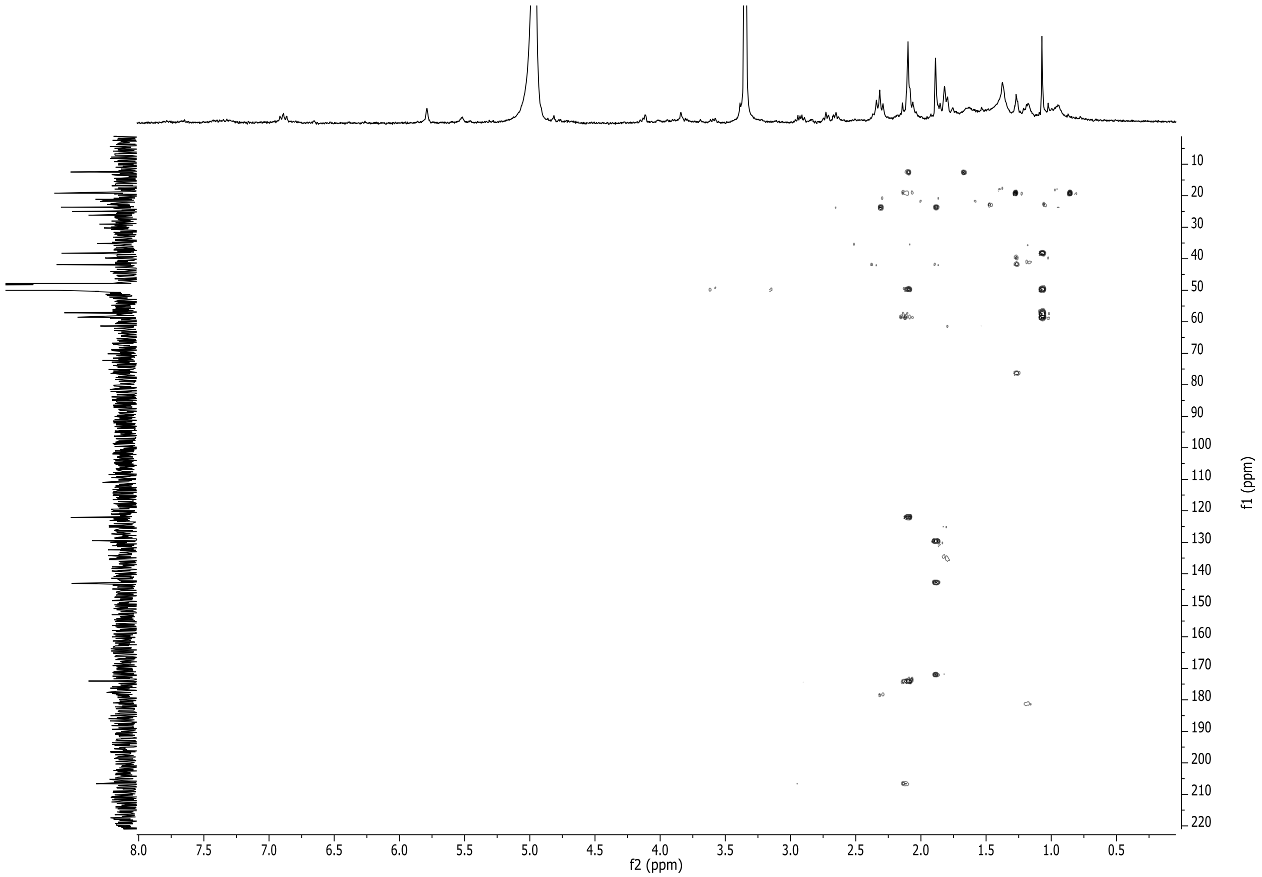

Figure S8: HMBC spectrum for heydenoic acid A (**1**) in MeOD

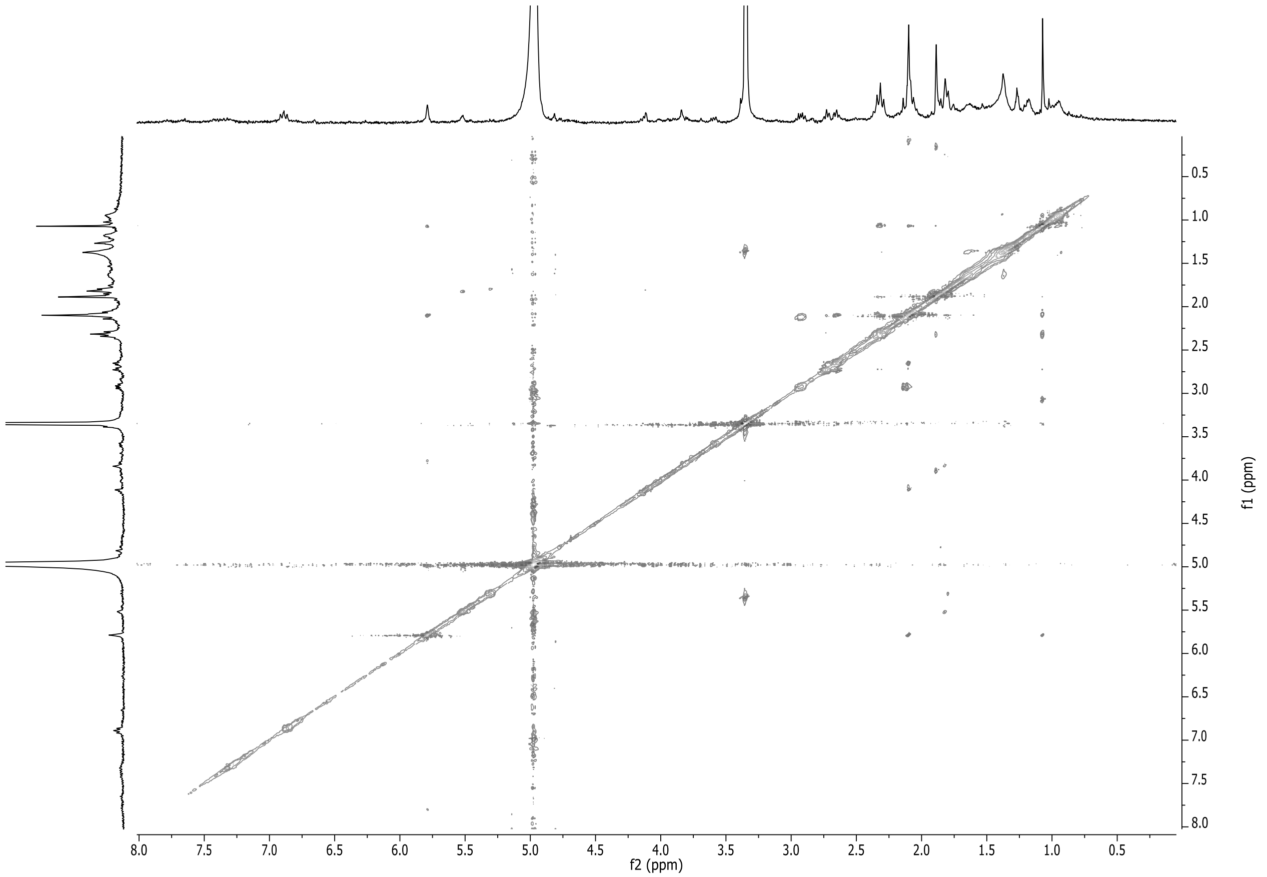

Figure S9: NOESY spectrum for heydenoic acid A (**1**) in MeOD

1D and 2D NMR spectra of heydenoic acid B (2)

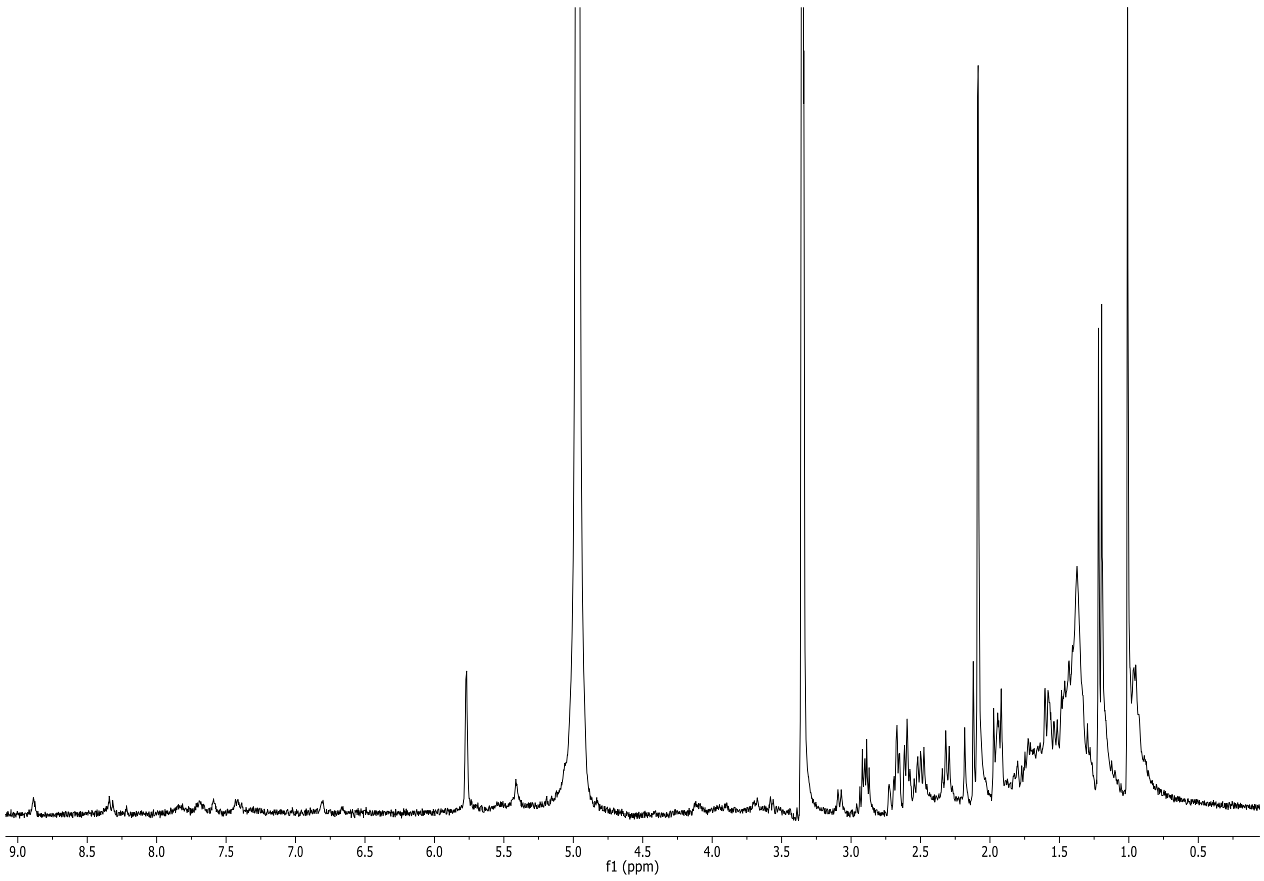

Figure S10: ^{1}H NMR of heydenoic acid B (**2**) (300 MHz, in MeOD)

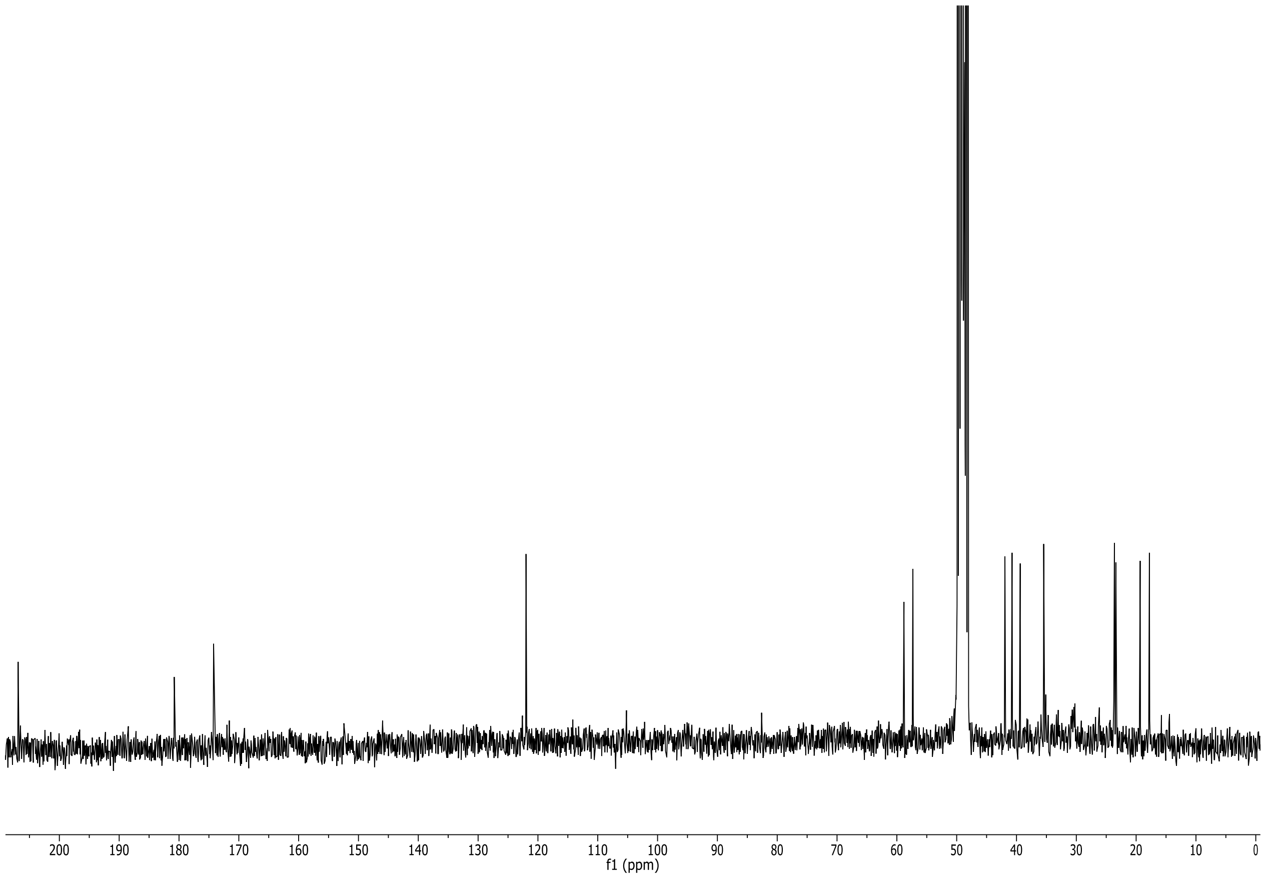

Figure S11: ^{13}C NMR of heydenoic acid B (**2**) (75 MHz, in MeOD)

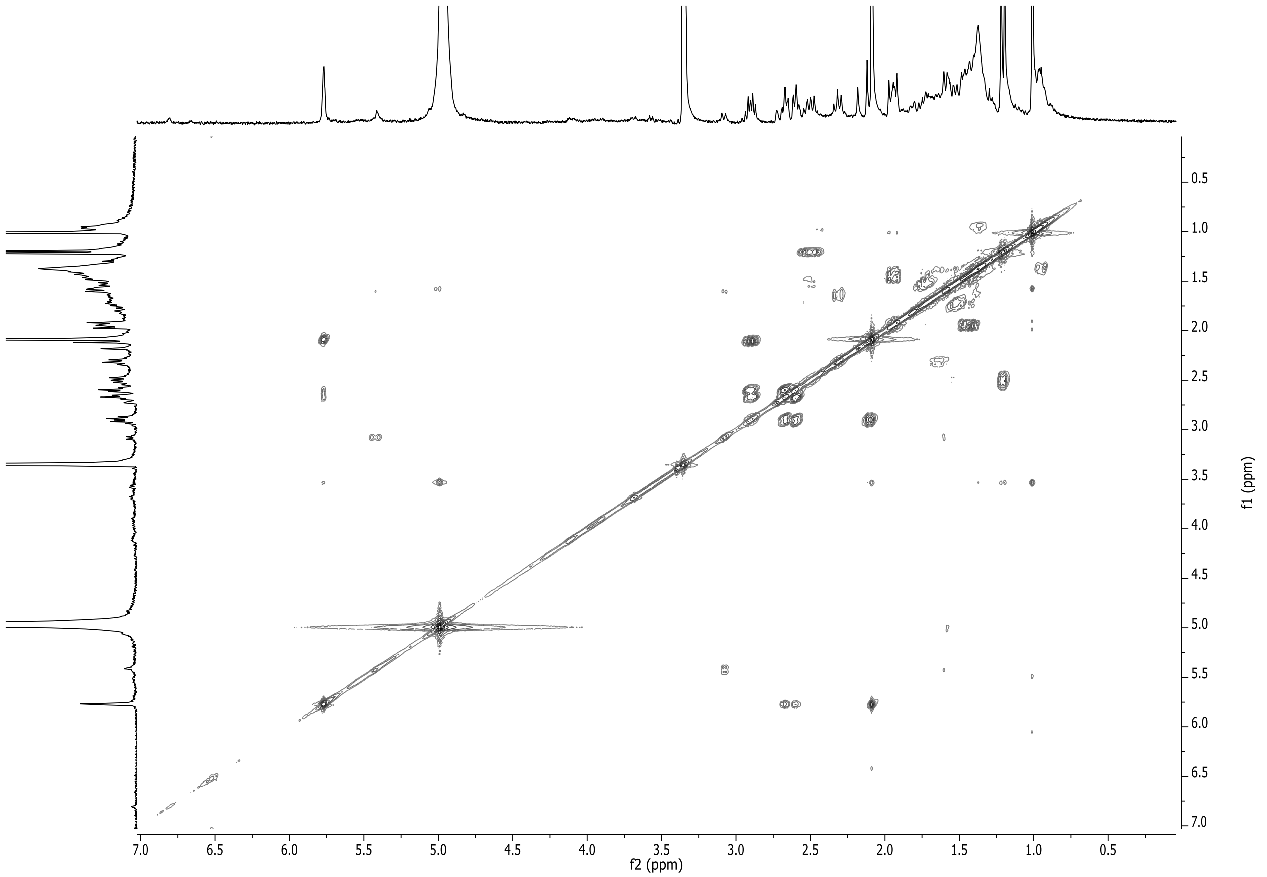

Figure S12: $^{1}H,^{1}H$-COSY spectrum for heydenoic acid B (**2**) in MeOD

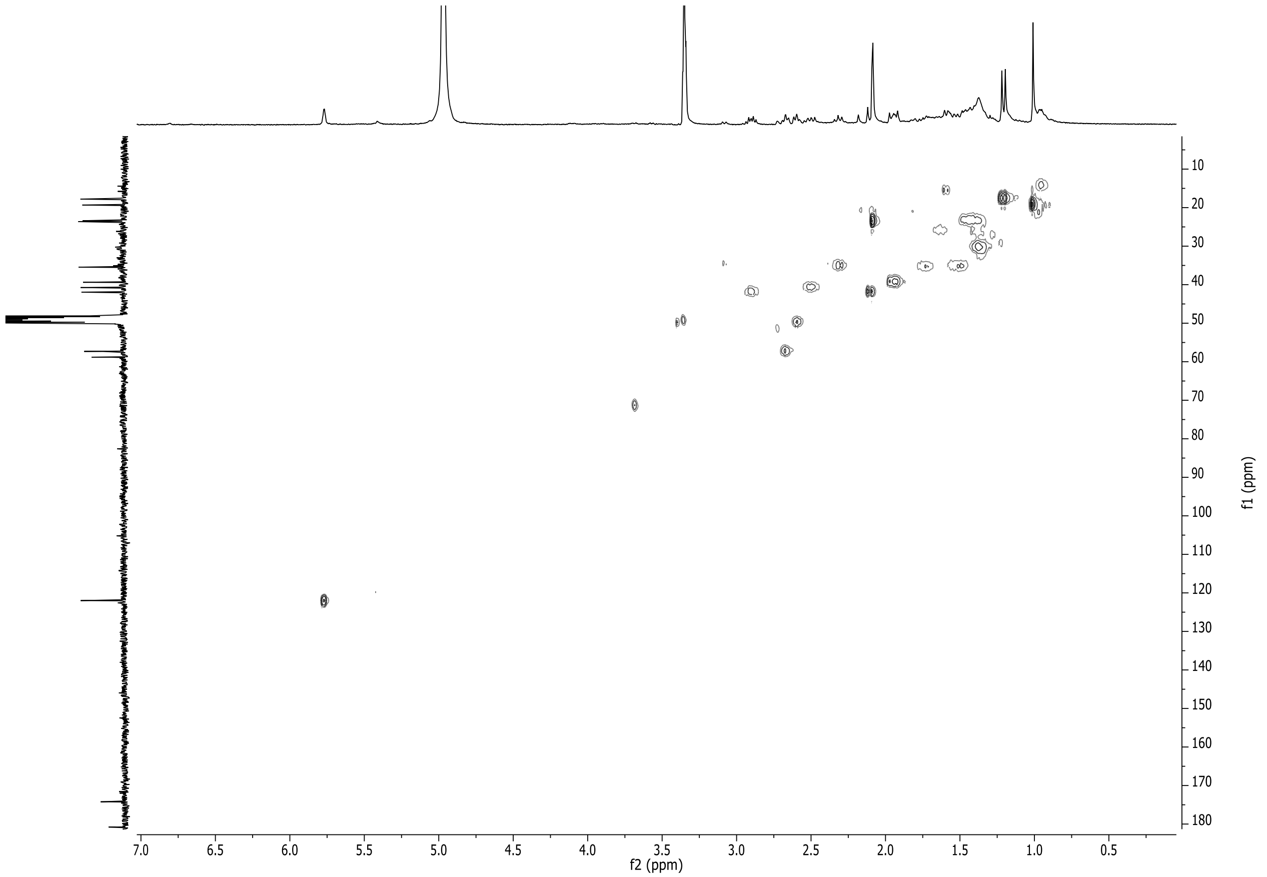

Figure S13: HSQC spectrum for heydenoic acid B (**2**) in MeOD

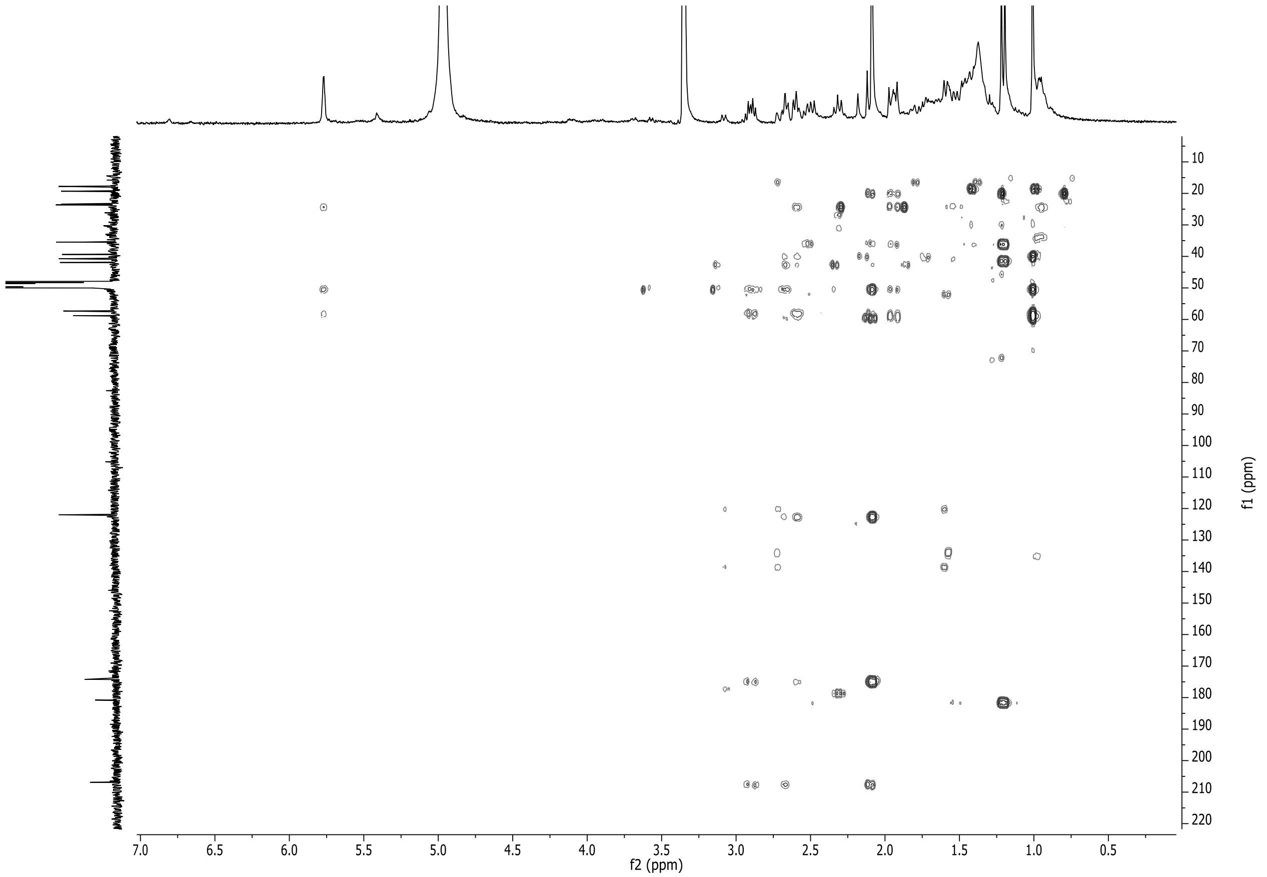

Figure S14: HMBC spectrum for heydenoic acid B (**2**) in MeOD

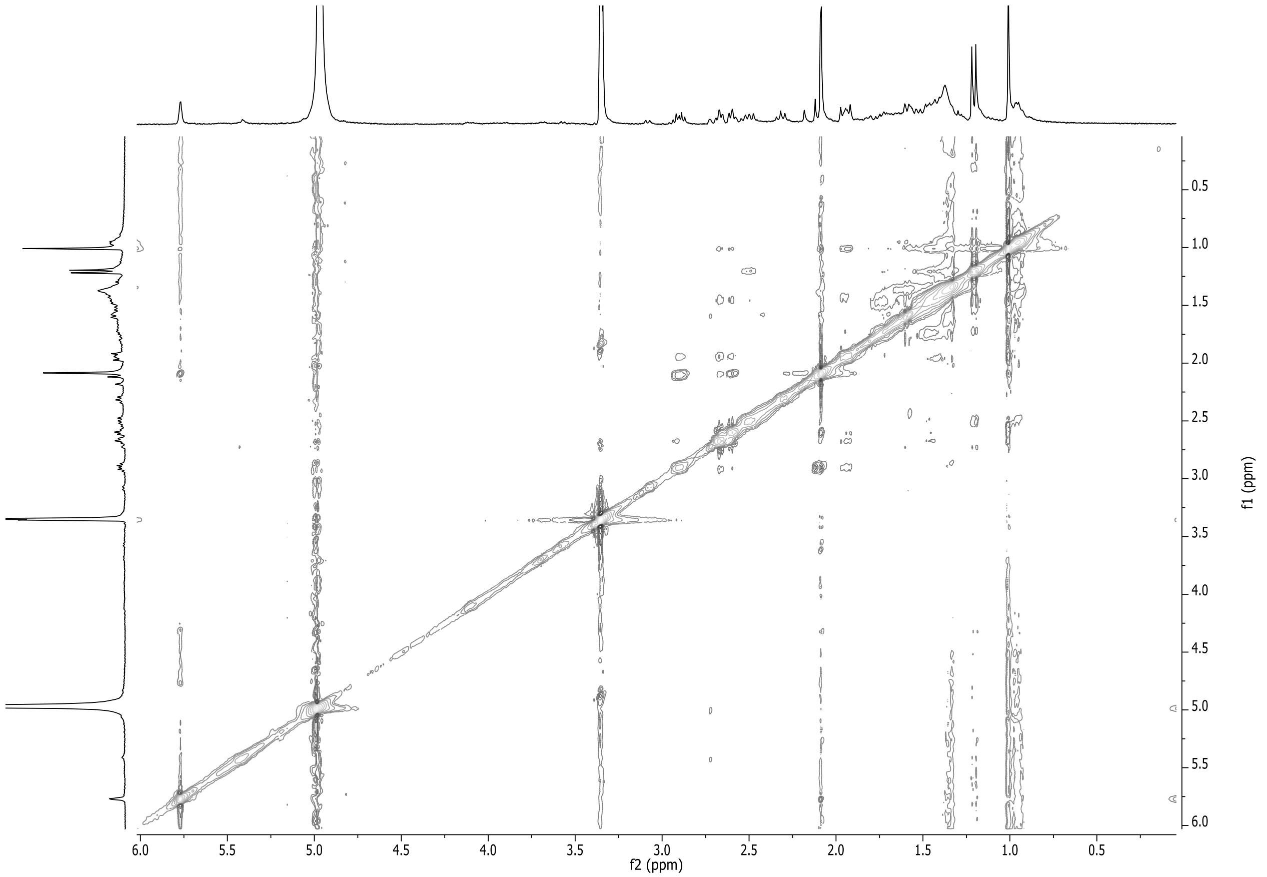

Figure S15: NOESY spectrum for heydenoic acid B (**2**) in MeOD
